# Targeting epigenetic regulators to overcome drug resistance in cancers

**DOI:** 10.1038/s41392-023-01341-7

**Published:** 2023-02-17

**Authors:** Nan Wang, Ting Ma, Bin Yu

**Affiliations:** Institute of Drug Discovery & Development, School of Pharmaceutical Sciences, Zhengzhou University, Zhengzhou, 450001 China

**Keywords:** Drug discovery, Chemical biology

## Abstract

Drug resistance is mainly responsible for cancer recurrence and poor prognosis. Epigenetic regulation is a heritable change in gene expressions independent of nucleotide sequence changes. As the common epigenetic regulation mechanisms, DNA methylation, histone modification, and non-coding RNA regulation have been well studied. Increasing evidence has shown that aberrant epigenetic regulations contribute to tumor resistance. Therefore, targeting epigenetic regulators represents an effective strategy to reverse drug resistance. In this review, we mainly summarize the roles of epigenetic regulation in tumor resistance. In addition, as the essential factors for epigenetic modifications, histone demethylases mediate the histone or genomic DNA modifications. Herein, we comprehensively describe the functions of the histone demethylase family including the lysine-specific demethylase family, the Jumonji C-domain-containing demethylase family, and the histone arginine demethylase family, and fully discuss their regulatory mechanisms related to cancer drug resistance. In addition, therapeutic strategies, including small-molecule inhibitors and small interfering RNA targeting histone demethylases to overcome drug resistance, are also described.

## Introduction

Increasing evidence has suggested that the incidence of multiple cancers has been rising year by year in the world.^1^ Although great progress has been made in the treatment of cancer, it is still the main cause of death. Chemotherapy, radiotherapy, immunotherapy, surgical resection, and targeted cancer therapies are usually used in cancer treatment.^2–5^ Generally, good therapeutic effects can be achieved in the early stage of cancer, but after long-term treatment with chemotherapeutic drugs, tumor cells could develop drug resistance. Drug resistance is closely related to poor prognosis and cancer recurrence, which is one of the main reasons for cancer treatment failure. Therefore, it is desirable to elucidate the resistance mechanisms in tumor cells during treatment.

**Fig. 1:**
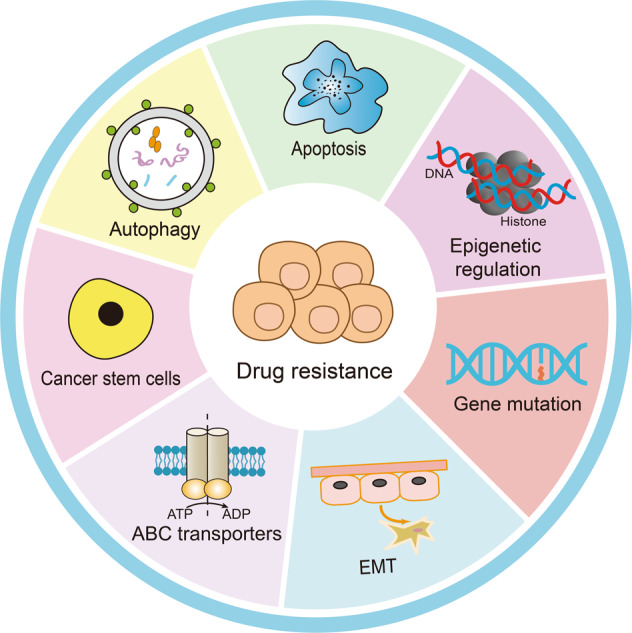
Common drug resistance mechanisms. There are seven main reasons that can lead to cancer drug resistance, including overexpressed ABC transporters, CSCs, autophagy, apoptosis, gene mutations, EMT, and epigenetic regulation

To date, there are many identified drug resistance mechanisms (Fig. 1). As a member of ATP-binding cassette (ABC) transporters, P-glycoprotein (P-gp) can promote the efflux of drugs and make the drugs lose their therapeutic effect, inhibition of P-gp can significantly reverse drug resistance.^6–10^ Additionally, cytoprotective autophagic response often counteracts apoptosis triggered by anticancer drugs, potentially contributing to acquired drug resistance.^11,12^ Notably, it is generally believed that eliminating cancer stem cells (CSCs) and reversing epithelial-mesenchymal transition (EMT) are also effective means to overcome drug resistance.^13–16^ Additionally, gene mutations also contribute to drug resistance, especially the resistance to tyrosine kinase inhibitors. EGFR mutation can usually lead to resistance to tyrosine kinase inhibitors, and Bruton’s tyrosine kinase mutation can also result in resistance to ibrutinib.^17–19^

**Fig. 2:**
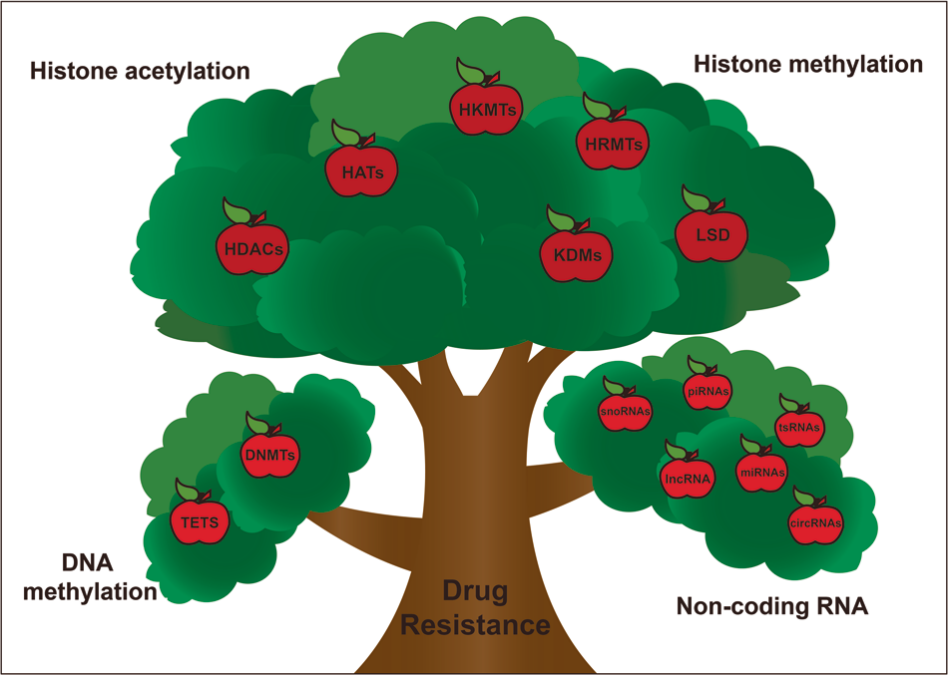
Epigenetic regulation mechanisms of drug resistance. Non-coding RNAs, DNA methylation, and histone modifications including histone acetylation and methylation play important roles in tumor resistance. DNA methylation is maintained by DNA methyltransferases and DNA demethylases, histone acetylation is regulated by histone acetyltransferases and histone deacetylases, and histone methylation is maintained by histone demethylases and histone methyltransferases

In addition to these factors mentioned above, epigenetic regulation is also important in mediating drug resistance (Fig. 2). Epigenetic modifications refer to the heritable changes in gene expression without changes in DNA sequence, including DNA methylation, histone modification, X-chromatin remodeling, non-coding RNA, nucleosome localization and genomic imprinting.^20,21^ Of note, modifications of DNA and histone not only affect the function of transcription factors, but also tightly associate with other epigenetic modifications such as chromatin remodeling and non-coding RNAs to co-regulate neoplastic processes.^22,23^ Generally, DNA methylation often affects gene expression, transcription, and activity. Under the action of DNA methyltransferase, a methyl group is covalently added to the C-5 position of the DNA cytosine ring, and hypermethylation of gene promoters usually leads to transcriptional inhibition, resulting in decreased gene expression.^24–26^

Similarly, covalent histone modification is also an important epigenetic model, which includes acetylation, phosphorylation, methylation, ADP ribosylation, ubiquitination, and citrullination, etc.^27–31^Among these modifications, most of the studies have focused on the acetylation, methylation, and phosphorylation. Numerous studies have shown that covalent post-translational modification of histone tails is critical to the occurrence and development of cancer, including histone demethylases, histone methyltransferases, histone deacetylases, histone acetyltransferases (HATs), and ADP ribosyltransferases.^32–35^ For example, it has been shown that histone acetylation is closely associated with gene transcription and the alterations of histone acetylation are tightly associated with cancer phenotypes in multiple cancers.^36,37^ Furthermore, cancer cells can produce significant resistance to chemotherapeutic drugs through epigenetic changes, especially abnormal modification of histone or genomic DNA.^38,39^ Their dysregulation usually results in the activation of oncogenes or the inactivation of tumor suppressor genes as well as the dysfunction of many signaling pathways. Therefore, targeting epigenetic modifications could be a promising strategy to overcome drug resistance. Herein, in this review, we first summarize the roles of different epigenetic regulators in tumor resistance. We further summarize the classification and functions of histone demethylases and describe the relationship between histone demethylases and cancer drug resistance in detail.

## DNA methylation and drug resistance

In eukaryotes, DNA methylation is an enzymatic reaction catalyzed by a series of DNA methyltransferases, in which a methyl group is covalently added to the 5-carbon of the cytosine ring within a CpG dinucleotide.^23^ Studies have shown that the activity of some tumor suppressor genes is suppressed due to DNA methylation, which is the basic pathogenesis of multiple cancers. The retinoblastoma tumor suppressor (RB1) was the first identified tumor suppressor gene that is hypermethylated in tumor tissues.^40^ In breast and ovarian cancers, the DNA repair gene BRCA1 is also silenced because of hypermethylation.^41^

There are five members in the DNA methyltransferase family, namely DNMT1, DNMT2, DNMT3A, DNMT3B, and DNMT3L. Among them, only three have catalytic methyltransferase activity.^24,42^ Considered a maintenance methyltransferase, DNMT1 preferentially methylates hemimethylated DNA and is responsible for replicating parental DNA methylation patterns to newly synthesized DNA daughter strands.^43^ On the contrary, regarded as de novo methyltransferases, DNMT3A and DNMT3B are more biased towards methylating unmethylated CpG dinucleotides.^44^ The remaining two DNA methyltransferases are generally thought to lack cytosine methyltransferase activity, although DNMT3L can increase the binding capacity of DNMT3A and DNMT3B to the methyl donor S-adenosyl-L-methionine (SAM) to increase their activity.^45^

**Fig. 3:**
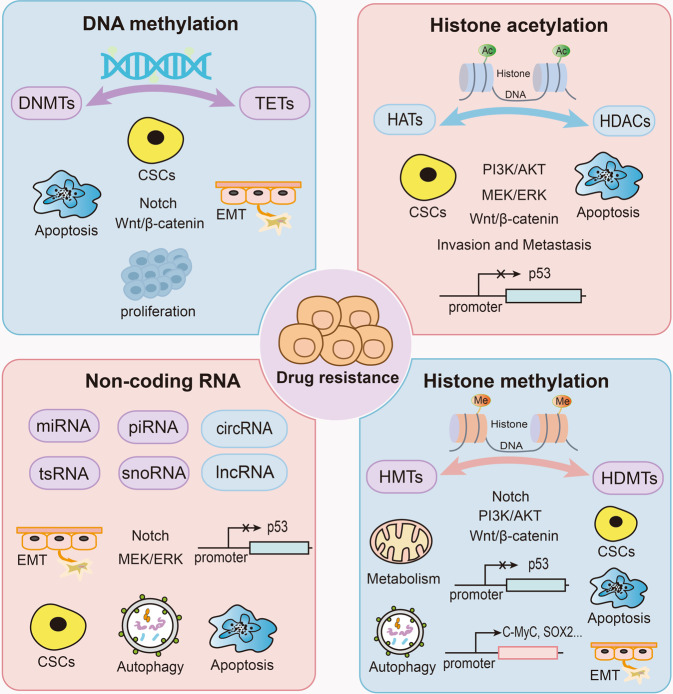
The roles of different epigenetic modifications in tumor resistance. DNA methylation is co-regulated by DNA methyltransferases and DNA demethylases, mainly influencing apoptosis, stemness, EMT, and cell proliferation through Notch and Wnt/β-catenin signaling pathways to modulate drug resistance. Non-coding RNAs mainly include four small nucleotides and two large nucleotides, which promote or inhibit tumor resistance. Similarly, histone methylation and acetylation are histone modifications. They keep gene expressions in balance through histone demethylases and methyltransferases, histone acetyltransferases and histone deacetylases, respectively, and function as a double-edged sword in cancer resistance

Studies have shown that DNA methyltransferase is closely related to tumor resistance (Fig. 3). CSCs are key factors in cancer resistance, and under the regulation of DNMT1, brain-expressed X-linked protein 1 (BEX1) can activate Wnt/β-catenin signaling to maintain the self-renewal capacity of liver stem cells.^46^ In glioma stem cells (GSCs), the interaction between CD133, a marker of CSCs, and DNMT1 inhibited the nuclear translocation of DNMT1 and maintained the self-renewal and tumorigenesis of GSCs, thus promoting GSCs resistant to the chemotherapeutic agent temozolomide.^47^ In addition, DNMT1-induced hypermethylation of the miR-34a promoter region mediated its silence and aberrant activation of the Notch pathway, while treatment with decitabine, a small-molecule inhibitor of DNMT1, can increase the sensitivity of pancreatic cancer cells to sorafenib.^48^ DNA methyltransferases DNMT3A and DNMT3B are overexpressed in Rhabdomyosarcoma and tamoxifen-resistant breast cancers, which indicated that targeting DNA methyltransferases could serve as a potential strategy for radiosensitization and chemosensitization.^49,50^ Moreover, DNMT3B was also increased in sorafenib-resistant cells, and DNMT3B inhibition by nanaomycin A significantly increased the sensitivity of HCC cells to sorafenib.^51^ Similarly, DNMT3B mRNA expression levels are also negatively correlated with decitabine sensitivity in pancreatic cancers.^52^ However, Jia Yu et al. found that triple-negative breast cancers with high expression of DNMTs were more sensitive to decitabine treatment.^53^ And in ovarian cancers, decitabine sensitivity also correlated more closely with high expression of DNMT1.^54^ Taken together, this evidence indicates that DNA methyltransferases play a dual role in tumor resistance.

In addition to DNA methyltransferases, DNA demethylases also play an important role in drug resistance (Fig. 3). Ten-eleven translocations (TETs) are a family of DNA demethylases that work with DNMTs to maintain the balance of DNA methylation, which consist of TET1, TET2, and TET3.^55^ As a founding member of the TET family, TET1 catalyzes the oxidation of methyl cytosine to hydroxymethyl cytosine, and then lead to further hydroxylation and oxidation to remove the methyl group from methylated cytosine.^55,56^ In 5-fluorouracil-resistant colon cancer cells, TET1 binds to the NADPH oxidase DUOX2 promoter and induces its transcription by regulating its methylation, thereby promoting EMT and increasing ROS level.^57^ Besides, enriched in the promoter region of CRABP2, TET1 can increase its expression, thereby inhibiting Bax-dependent apoptosis to induce oxaliplatin resistance in gastric cancer cells.^58^ Interestingly, TET1 knockdown can cause lung cancer cells resistant to gefitinib.^59^ Similarly, the knockdown of TET2 triggered resistance in BRCA2 deficient cells to multiple poly (ADP-ribose) polymerase (PARP) inhibitors and cisplatin.^60^ And loss of TET2 also reduced ERα expression, thereby causing endocrine resistance in breast cancer cells.^61^ As a tumor suppressor, inactivation of the Retinoic acid receptor-β (RARβ) gene by methylation of its promoter contributes to tumorigenesis and drug resistance in various cancers,^62^ whereas TET2 overexpression can reverse its reduction and increase the sensitivity of squamous cell lines to All-*trans*-retinoic acid (ATRA).^63^

Compared with the above two DNA demethylases, TET3 has been less studied in tumor resistance. Negatively correlated with TLX and positively correlated with laminin-integrin α6, upregulation of TET3 is able to increase the levels of tumor suppressor genes, thereby inhibiting the growth and self-renewal ability in glioblastoma stem cells.^64,65^ Regulation of stemness is perhaps responsible for the reduction of glioblastoma resistance induced by TET3, however, the underlying mechanisms of TET3-regulated drug resistance in other cancers require further investigation. More importantly, in addition to the roles in regulating tumor development by influencing gene expression, DNA methylation can interact with other epigenetic modifications to synergistically regulate chromatin formation, including histone modification, nucleosome localization, etc. Related contents have been described in detail in previously published article.^24^

Up to now, many small-molecule inhibitors targeting DNA methylation have been developed. Among these, DNMT inhibitors azacytidine, guadecitabine (SGI-110), and decitabine have already entered clinical trials.^66,67^ In 2018, a phase III clinical trial of azacitidine has been completed to evaluate its safety profile and to determine whether azacitidine can cure patients with acute myeloid leukemia (AML), chronic myelomonocytic leukemia (CMML) or myelodysplastic syndromes (MDS) following allogeneic (donor) stem cell transplantation (ClinicalTrials.gov Identifier: NCT00887068). In the same year, a phase II clinical trial of azacitidine for injectable suspension has been completed to explore its effect on patients with prostate cancer (ClinicalTrials.gov Identifier: NCT00384839). In this study, a total of 35 patients with hormone-refractory prostate cancer received azacitidine for 5 consecutive days of each 28-day cycle and response was assessed after a minimum of 2 cycles. In addition, its roles in head and neck squamous cell carcinoma have also been studied in a phase I trial (ClinicalTrials.gov Identifier: NCT02178072). Decitabine also inhibited DNMT1 activity, and its effect on multiple cancers has also been studied in patients with AML (ClinicalTrials.gov Identifier: NCT00416598), refractory diffuse-large B-cell lymphoma (ClinicalTrials.gov Identifier: NCT03579082), follicular thyroid cancer (ClinicalTrials.gov Identifier: NCT00085293), etc. However, although several clinical trials have been carried out for these two drugs, their impact on drug resistance is rarely involved. Of note, although most of the clinical trials of SGI-110 has been focused on AML, a phase I clinical trial of SGI-110 was initiated to evaluate the efficacy for restoring cisplatin sensitivity in refractory germ cell tumor (ClinicalTrials.gov Identifier: NCT02429466), indicating that targeting DNA methylation is an effective therapeutic measure to overcome tumor resistance. A total of 15 subjects were enrolled in this study and SGI-110 was subcutaneously given daily, 30 mg/m^2^ on days (1–5) followed by cisplatin 100 mg/m^2^ on day 8 every 4 weeks.

## Non-coding RNA and drug resistance

As another important part of epigenetics, non-coding RNAs (ncRNAs) have been recognized as key regulators of virtually every cellular process which is closely associated with gene expression. In addition, they act as oncogenes or tumor suppressors in various cancers as well.^68^ Generally, non-coding RNAs can be divided into small nucleotides and large nucleotides (Fig. 3), and small ncRNAs are further divided into small nucleolar RNAs (snoRNAs), P-element induced WImpy testis (PIWI)-interacting RNAs (piRNAs), miRNAs, and transfer RNA (tRNA)-derived small RNAs (tsRNAs).^69,70^

MiRNAs can bind with complementary sequences in the 3’ untranslated region (3’UTR) of target mRNAs, leading to translational repression or degradation of the target mRNA.^71^ So far, a large number of miRNAs have been found, and different miRNAs have different roles in tumors. For instance, miR-155,^72^ miR-21^73^ and miR-10b^74^ can act as oncogenes and promote tumor initiation and progression, whereas miRlet-7,^75^ miR-15a, miR-16-1^76^ and miR-34a^77^ can function as tumor suppressors. tsRNAs are derived from mature transfer RNAs (tRNAs) and have similar functions to miRNAs.^68^ TRF/miR-1280 has been reported to inhibit epithelial-mesenchymal transformation and CSCs by inhibiting the Notch pathway in colorectal cancer.^78^ Moreover, aberrant expression of piRNAs is strongly associated with the development of human malignancies by loading onto members of the PIWI subfamily of argonaute proteins to silence transposons.^79–81^ PiRNAs can also act as promoters or suppressors of tumor development by regulating DNMT expression and DNA methylation.^82^ Mainly found in the nucleolus, small nuclear RNAs (snoRNAs) not only direct posttranscriptional modifications of ribosomal RNAs and some spliceosomal RNAs, but also participate in the nucleolytic processing of original rRNA transcripts.^83,84^ SnoRNAs can be divided into two main families: C/D box snoRNAs (SNORDs) and H/ACA box snoRNAs (SNORAs), and jointly regulate the development of cancer.^85,86^ SNORA42, an H/ACA box snoRNA, is overexpressed in non-small-cell lung cancer and its expression is negatively correlated with clinical survival.^87^ In contrast, the expression of C/D box snoRNAs U50 has been reported to be downregulated in breast and prostate cancers.^88,89^

Large ncRNAs include circular RNAs (circRNAs) and long non-coding RNAs. CircRNAs are single-stranded RNAs with covalently closed circular structure, which are produced by back-splicing process of linear precursor RNAs.^90,91^ Acting as oncogenic factors or tumor suppressors, circRNAs are able to directly bind to proteins and interact with miRNAs to regulate cancer initiation.^92,93^ Likewise, long non-coding RNAs are able to bind to both proteins and DNA to exert either tumor-promoting or tumor-suppressive effect. For example, MEG3 can upregulate p53 and promote the binding of p53 to the promoter of growth differentiation factor 15 (GDF15), thereby inhibiting tumor growth.^94^ On the contrary, as an oncogene, lncRNA HOX transcript antisense RNA overexpression can increase the metastatic and invasive abilities in various tumors.^95,96^

The relationship between non-coding RNAs and tumor resistance has been reported (Fig. 3). Recent studies have described the effects of non-coding RNAs on drug resistance in colorectal and lung cancer, including lncRNA, miRNA, and circRNAs, indicating that ncRNAs can be used as biomarkers to predict drug resistance.^97–99^ Fatemeh Najafi et al. also focused on the involvement of miR-424 and miR-631 in the regulation of tumor resistance and sensitivity.^100^ EMT is an important mechanism by which tumors develop drug resistance, and HaShem khanbabaei et al. summarized the relationship between non-coding RNAs and EMT in cancers, suggesting that ncRNAs might serve as important regulators in tumor resistance.^70^ In addition to regulating EMT, non-coding RNAs also participate in the development of drug resistance by regulating CSCs, apoptosis, and autophagy.^101^ More interestingly, evidences have indicated that piRNAs may act as a double-edged sword in tumor resistance. Piwi-interacting RNA 1037 could enhance cisplatin resistance in oral squamous cell carcinoma (OSCC) cells by inhibiting cell apoptosis.^102^ Conversely, Piwi-interacting RNA piR-39980 induced intracellular doxorubicin accumulation, DNA damage, and cell apoptosis, thereby increasing fibrosarcoma sensitivity to doxorubicin.^103^

Additionally, the roles of lncRNA in drug resistance have also been studied extensively. For example, lncRNA Miat could promote the resistance of stem-like medulloblastoma (MB) cells to radiotherapy by downregulating p53 signaling pathway and reducing radiation-induced cell death.^104^ lncRNA MCF2L-AS1 activated the IGF2/MEK/ERK pathway by interacting with insulin-like growth factor-2 mRNA binding protein 1 (IGF2BP1), and knockdown of MCF2L-AS1 increased the sensitivity of ovarian cancer cells to cisplatin.^105^ Combined with miR-4496, AC116025.2 promoted the resistance of esophageal cancer cells to 5-FU by reducing cell apoptosis.^106^ Importantly, LncRNA can interact with LSD1 to regulate drug resistance. Overall, non-coding RNAs are not represented as unimportant, although these non-coding RNAs cannot be translated into proteins, they can interact with DNA, RNA, and proteins to participate in various of cellular activities and modulate tumor resistance.

Based on the characteristics of non-coding RNAs, using ncRNA or directly targeting ncRNA may become an effective strategy for the precise treatment of cancer patients. Up to now, many studies about non-coding RNA have entered clinical trials.^107^ And most clinical trials aim to validate the roles of non-coding RNAs as cancer biomarkers, such as long non-coding RNA HOTAIR as a biomarker in thyroid cancer (ClinicalTrials.gov Identifier: NCT03469544) and long non-coding RNAs WRAP53 and UCA-1 as biomarkers in hepatocellular carcinoma (ClinicalTrials.gov Identifier: NCT05088811). However, the role of non-coding RNAs in drug resistance has not been clinically studied.

## Histone modifications and drug resistance

### Histone acetyltransferases (HATs)

Many lysine residues on histone tails are capable of acetylation, and studies have shown that histone acetylation contributes to cancer development by regulating intracellular pH and affecting the gene transcriptional activity and chromatin structure.^108^ Histone acetylation is a dynamic process, which is regulated by two enzymes with opposite functions: HATs and histone deacetylases (HDACs), and the imbalance often leads to the occurrence of tumors.^109^ HATs are able to catalyze the transfer of an acetyl group from the donor acetyl coenzyme A to histone lysine side chains, eliminating the positive charge of lysine and thus unfolding the local structure of chromatin.^110^ HAT is mainly composed of three families located in the nucleus: the MYST family (Moz-Ybf2/Sas3-Sas2-Tip60), the p300/CREB-binding protein family (CBP/CREBBP), and the GCN5-related N-acetyltransferases family (GNAT).^111–113^ In addition, there is also a histone acetyltransferase Hat1 in the both nucleus and cytoplasm, and it is mainly responsible for acetylating newly synthesized histone H3 and H4.^114^

As a member of the MYST family, MYST1, also named hMOF, can acetylate H4K16 and is abnormally expressed in many cancers, including gastric cancer, breast cancer, non-small cell lung cancer, etc.^115–117^ TIP60, another member of MYST family, can also acetylate histone H2A, H3, and H4 and a variety of non-histones, such as p53, STAT3, NF-κB, etc.^118,119^ CBP/p300 can inhibit H3K27 acetylation to block estrogen receptor *α* in breast cancer, and treatment with the CBP/p300 inhibitor A-485 specifically reduced CBP/p300 mediated histone acetylation and led to growth arrest of cells by activating the autophagy pathway in NSCLC.^120,121^ Moreover, the upregulation of GCN5 is usually closely related to the poor prognosis of tumors.^122^ In prostate cancer, GCN5-mediated acetylation of LIFR promoted its homodimerization, subsequently promoted LIFR-S1044 phosphorylation, thus activating AKT signaling.^123^ Interestingly, these HATs can also affect the activity of histone methyltransferases in vitro.^124^

Many studies have reported the roles of HATs in drug resistance (Fig. 3). High expression of histone acetyltransferase 1 (HAT1) can enhance the resistance of castration-resistant prostate cancer and pancreatic cancer to enzalutamide and gemcitabine.^125,126^ Mechanistically, HAT1 can promote PVT1 transcription and improve the stability of EZH2 protein to increase drug resistance.^126^ Besides, inhibition of lysine acetyltransferase 6 A (KAT6A), a member of MYST family, can induce apoptosis and enhance the sensitivity of ovarian cancer cells to cisplatin.^127^ KAT2A can also contribute to tamoxifen resistance in breast cancer by reducing p53 levels, and KAT2A knockdown sensitizes prostate cancer cells to abiraterone.^128,129^ Interestingly, in addition to these HATs, P300 has been reported to be able to regulate drug resistance in tumors as a double-edged sword. In pancreatic cancer, loss of P300 could mediate Wnt/β-catenin independent tumor growth, thus leading to resistance to PORCN inhibition.^130^ P300 inhibition also rendered bladder cancer cells resistant to doxorubicin.^131^ On the contrary, P300 encoding gene EP300 was able to combine with the COL1A2 promoter to activate its expression, thus promoting apatinib resistance in GC cells.^132^ And upregulation of P300 promoted pERK1/2 rebound and subsequent resistance of melanoma cells to BRAF inhibitors.^133^ In conclusion, most HATs can promote drug resistance and act as therapeutic targets to prevent or overcome drug resistance in tumors.

Therefore, targeting HATs to develop small-molecule inhibitors holds great promise as a promising measure to reverse drug resistance in the treatment of cancer. Many previously developed HAT inhibitors have been summarized in other articles.^134,135^ However, few inhibitors have entered clinical trials and the existed inhibitors mainly target CBP/P300. Phase I/II clinical studies of CBP/P300 inhibitor CCS1477 were initiated for the treatment of hematologic malignancies, prostate cancer, breast cancer, and NSCLC (ClinicalTrials.gov Identifier: NCT03568656; NCT04068597). In 2022, a phase I clinical trial was started to evaluate the safety and maximum tolerated dose of EP31670, a dual inhibitor of BET and CBP/P300, in advanced solid tumors (ClinicalTrials.gov Identifier: NCT05488548).

### Histone deacetylases (HDACs)

The balance between histone deacetylases and acetyltransferases is a key regulatory mechanism of gene expression and is involved in various activities and disease occurrence. Contrary to the HATs, histone deacetylases can remove acetyl groups from histone or non-histone lysine residues, thus concentrating chromatin and weakening gene transcription activity.^136,137^ According to the structure and sequence similarities, histone deacetylases can be grouped into four families comprising a total of 18 isozymes: Class I, II, III, and IV. Class I RPD3-like proteins consist of HDAC1, 2, 3, 8, which mainly located in the nucleus. Class II HDACs consist of class IIa (HDAC4, 5, 7, and 9) and class IIb (HDAC6 and 10) which can shuttle between cytoplasm and nucleus to regulate the cytoplasmic substrate. As nicotinamide adenine dinucleotide (NAD^+^)-dependent deacetylases, Class III HDACs mainly include Sirtuin (SIRT)1-7, which are homologous to the Sir2 protein in yeast. And located in the nucleus, the single member of the class IV HDACs, HDAC11, has gained prominence in epigenetics.^107,111^ Targeting histones or non-histone proteins, HDACs are able to act as both tumor promotors and suppressors, and dually regulate cancer progression by influencing cellular activities such as stemness, proliferation, apoptosis, differentiation, angiogenesis, migration, and invasion.^111,138,139^

Generally, high expression of HDAC will promote tumor resistance and amongst of these HDACs, histone deacetylase HDAC6 is widely concerned in regulating drug resistance.^140^ In NSCLC and melanoma, HDAC6 can enhance the stability of EGFR and tubulin β3 and reduce apoptosis, subsequently resulting in tumor resistance.^141,142^ HDAC1/2/6 induced deacetylation of specificity protein 1 (Sp1) promoted CSC-like cell proliferation, thus enhancing the resistance of gliobalastoma to temozolomide.^143^ Notably, ubiquitin-specific peptidase 10 (USP10) was also able to interact with HDAC6 and increase its stabilization, further increasing cisplatin resistance in NSCLC.^144^ In addition to HDAC6, SIRT7 can deacetylate p53 and reduce the sensitivity of hepatocellular carcinoma to adriamycin.^145^ SIRT1 increased β-catenin expression and nuclear translocation, thus enhancing the resistance of colorectal cancer (CRC) cells to radiotherapy.^146^ Surprisingly, SIRT3 and SIRT1 have also been reported to be tightly associated with insulin resistance.^147,148^ Significantly overexpressed in glioblastoma, the class I HDACs HDAC1/3/8 also contributed to temozolomide resistance.^149^ In summary, aberrant expression of histone deacetylases promotes the development of tumor resistance, and targeting histone deacetylases can limit the generation of drug resistance (Fig. 3).

Up to now, HDAC inhibitors have been extensively developed and become the focus of cancer treatment. Four HDAC inhibitors have been approved by the food and Drug Administration for clinical treatment, including romidepsin, belinostat, panobinostat, and vorinostat.^150^ In addition, many other HDAC is are undergoing clinical evaluation now, and the combination of HDAC is with mTOR inhibitors, EGFR inhibitors, PI3K inhibitors, and immune checkpoint inhibitors has also become an important part of clinical and preclinical studies.^151,152^

### Histone methyltransferases

Histone methyltransferases (HMTs), also known as protein methyltransferases (PMTs), can be divided into histone lysine methyltransferases (HKMTs or PKMTs) and histone arginine methyltransferases (HRMTs or PRMTs). Depending on the products generated, histone arginine methyltransferases can be further classified into three classes: class I includes PRMT1, PRMT2, PRMT3, PRMT4 (CARM1), PRMT6, and PRMT8, which primarily catalyze the formation of monomethyl arginine and asymmetric dimethylarginine; class II consists of PRMT5 and PRMT9, which mainly catalyze the formation of monomethyl arginine and symmetric dimethylarginine; class III only includes PRMT7 which is responsible for catalyzing the formation of monomethyl arginine.^153,154^ Arginine methylation usually occurs in histones H3R2/R17/R26 and H4R3, which exerts an activating effect on gene expression. And the abnormal expression of PRMTs is conducive to the occurrence, development, and invasion of various tumors, including lung cancer, breast cancer, colorectal cancer, bladder cancer, and leukemia.^155,156^ However, HKMTs can perform opposite functions when acting on different substrates. Methylation of H3K4, H3K26, H3K36, H3K79, and H4K12 is mainly involved in gene activation, while methylation of H3K9, H3K27, H3K56, H4K5, and H4K20 is related to gene silencing.^157^ Highly expressed in tumors, SUV39H1, SETDB2 and G9a mainly target H3K9 for methylation, and H3K4 is the methylated target of KMT2A-E and KMT7. KMT3A-G can methylate H3K36 and is overexpressed in multiple cancers. In addition, DOT1L is the only histone lysine methyltransferase responsible for H3K79 methylation which can promote the occurrence of leukemia. Notably, much attention has been paid to the fact that EZH2 is mainly involved in tumorigenesis by regulating histone H3 lysine 27 (H3K27) methylation, whose overexpression is associated with poor prognosis.^157–160^

Histone methyltransferases are closely related to drug resistance (Fig. 3). High expression of EZH2 can activate cell survival pathways to promote ovarian cancer resistance to cisplatin.^161^ EZH2 can also activate PI3K/AKT pathway, thereby leading to acquired resistance to gefitinib in NSCLC.^162^ On the contrary, EZH2 inhibitor GSK126 can increase the expression of MEIS1 and make CRC cells sensitive to oxaliplatin.^163^ Additionally, SETDB1 can interact with PELP1 and activate AKT, thereby promoting the resistance of breast cancer to tamoxifen.^164^ Leukemic stem cells have been identified as an important cause of TKI resistance, and inhibition of G9A can increase the expression of tumor suppressor gene SOX6, thereby significantly inhibiting the survival and self-renewal ability of leukemia stem cells.^165^ In addition to lysine methyltransferases, elevated histone arginine methyltransferase PRMT5 level is also related to the malignant progression of tumor, and PRMT5 inhibition by GSK3186000A sensitized human AML cell lines to PARP inhibition.^166^ Similarly, class I PRMT inhibitor MS023 can decrease the resistance of ovarian cancer cells to PARP inhibitor BMN-673.^167^ Taken together, histone methyltransferases hold great promise as targets for cancer therapy to overcome tumor resistance. Up to now, many inhibitors have been developed for these histone methyltransferases, and many phase I/II clinical trials have been carried out for different cancers.^168–170^ Among these inhibitors, most of them target EZH2. Notably, in these clinical trials, combination of histone methyltransferase inhibitors with other therapeutics has also received widespread attention, suggesting an important role for drug combinations in cancer treatment.

### Histone demethylases (HDMTs)

**Fig. 4:**
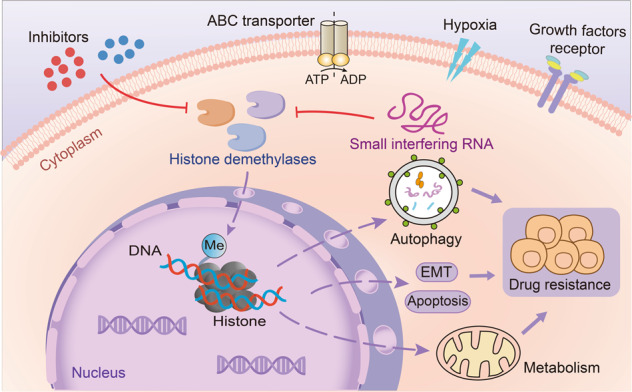
Regulation of histone demethylases in tumor resistance. Histone demethylases can contribute to the development of drug resistance by regulating gene transcription, promoting autophagy, reducing apoptosis, affecting cellular metabolic processes, and promoting epithelial-mesenchymal transition. Conversely, targeting histone demethylases by inhibitors or small interfering RNAs can reverse drug resistance

As a reversible dynamic regulatory process, abnormal histone methylation can directly or indirectly affect physiological and pathological processes. In this process, histone demethylases have gained much attention due to their important roles in tumor regulation. Many studies have revealed that histone demethylases (HDMTs) are tightly related to drug resistance and regulated by inhibitors and siRNA, histone demethylases can modulate drug resistance by affecting multiple activities including autophagy, EMT and metabolism (Fig. 4). Therefore, in this review, we summarize the classification and functions of histone demethylases, inhibitors in clinical trials, the roles of histone demethylases in tumor resistance and effective strategies targeting histone demethylases for reversing resistance.

#### Classification, function of histone demethylases

**Fig. 5:**
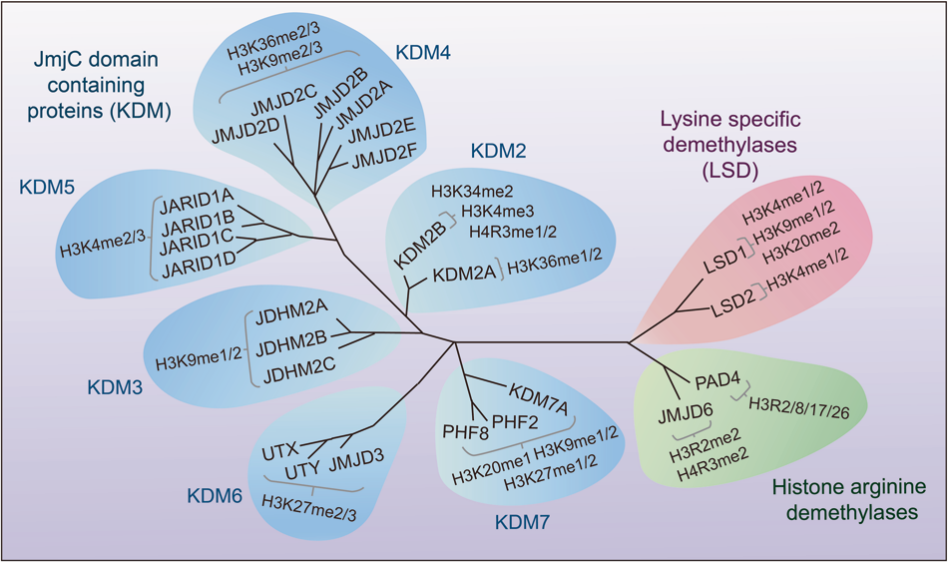
Classification of histone demethylases and their demethylation sites. There are three families of histone lysine demethylases (shown in different color): lysine-specific demethylase (LSD) family, JMJD family consisting of six families with 21 jmjC domain-containing proteins, and histone arginine demethylases. And different demethylases can demethylate different methylation sites of histones

Histone demethylases include histone lysine demethylases and histone arginine demethylases. A large number of histone lysine demethylases have been reported before, while histone arginine demethylases are relatively less reported (Fig. 5). There are three main families of histone demethylases: the lysine-specific demethylase (LSD) family, the Jumonji C(JmjC)-domain-containing demethylase (JMJD) family and the histone arginine demethylases.

##### The lysine-specific demethylase (LSD) family

**Fig. 6:**
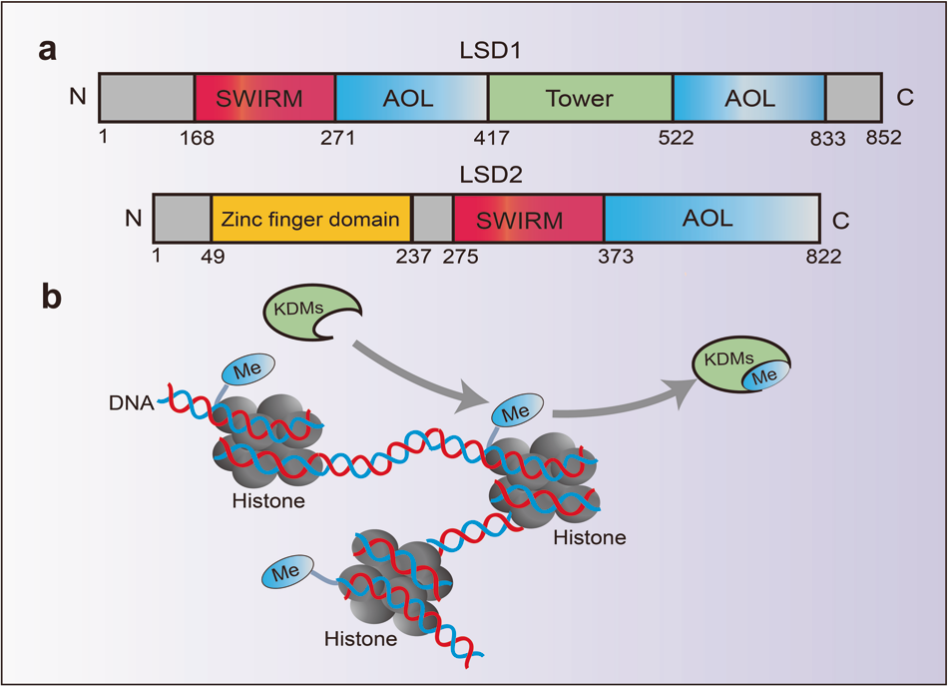
Structure domain of LSD family and catalytic mechanism. **a** Structure domain difference between LSD1 and LSD2. **b** Structure of histone and the demethylase catalytic process, every two molecules of histone H2A, H2B, H3, and H4 constitute a core protein octamer, and then about 146 bp of DNA is bound around the octamer to form a nucleosome, demethylases can function by removing methyl groups from histones

The lysine-specific demethylase (LSD) family consists of LSD1 and LSD2. Lysine-specific demethylase 1 (LSD1) is the first histone lysine demethylase which was found in 2004.^171^ LSD1, also known as KDM1A, BHC110, and AOF2, belongs to the flavin adenine dinucleotide (FAD) dependent ammonia oxidase superfamily.^172–174^ LSD1 is composed of three main domains: (1) the SWIRM domain, which is located at the N-terminal and responsible for the interaction with other proteins.^175^ (2) the Tower domain, which consists of two oppositely parallel α Helix composition and ensure the normal function of LSD1 demethylation.^176^ (3) the AOL domain, which is an amine oxidase-like domain locating at the C-terminal of LSD1 and is divided into two regions by the Tower domain, one is used for substrate binding and recognition, and the other is defined as the binding site of non-covalent FAD (Fig. 6).^177^

The combination of LSD1 with different co-factors or substrates can produce different functions. When interacted with CoREST,^178^ CtBP,^179^ NuRD,^180^ LSD1 can demethylate H3K4me1/me2, thus enhancing the expression of DNMTs and redemethylating DNA. In this way, it negatively regulates gene expression and inhibits gene transcription.^181^ However, in the presence of estrogen or androgen, LSD1 can demethylate H3K9 and activate gene expression.^182^ In addition, LSD1 can demethylate H3K20me2 and activate neural regulatory genes.^177^ At last, LSD1 can also interact with other proteins such as p53, p62, E2F1, and ERR non-histones and regulate their functions by demethylation.^183–187^

LSD2, also known as KDM1B and AOF1, a homolog of LSD1, is another FAD-dependent ammonia oxidase (Fig. 6).^188^ Both LSD2 and LSD1 have AOL and SWIRM domains, but the difference is that LSD2 contains N-terminal zinc finger domain, which is very important for its demethylation activity.^189^ LSD2 is an H3K4me1/me2 demethylase, it can inhibit the expression of p53 through H3K4me1/2 demethylation, promote the proliferation of colorectal cancer and inhibit its apoptosis.^190^ Besides, LSD2 can demethylate H3K4me1 and regulate the expression of TFPI-2, which plays a survival-promoting role in small cell lung cancer (SCLC).^191^ Besides, LSD2 also has E3 ubiquitin ligase activity, which can promote the proteasome degradation of O-GlcNAc transferase and inhibit the growth of lung cancer A549 cells.^192^ Compared with LSD1, the functions of LSD2 have been less studied, and more mechanisms need to be further explored.

##### The JmjC domain-containing proteins families

There are six families with 21 jmjC domain-containing proteins that can demethylate histone lysine and arginine.

*The FBXL (KDM2) family*: The FBXL family includes two proteins: FBXL11 and FBXL10.^193^ FBXL11, also known as JHDM1A or KDM2A, is the first discovered JmjC domain-containing demethylase, which can demethylate H3K36me1/me2 in a Fe (II) and α- ketoglutarate dependent manner.^194^ The high expression of KDM2A is influenced by multiple factors, including microRNA, inflammation, and hypoxia.^195,196^ Moreover, it plays an essential role in regulating the occurrence and development of gastric cancer and breast cancer.^197–199^ FBXL10, also named JHDM1B or KDM2B, has a specific H3K4me3 demethylase activity, which is able to inhibit ribosomal RNA and negatively regulate cell proliferation (Fig. 5).^200,201^ Furthermore, KDM2B can inhibit tumor growth in a p53-dependent manner.^202^ Besides, it can regulate and decrease the expression of p15INK4B by demethylating H3K4me2, so as to play a key role in the development and maintenance of leukemia.^203^

*The JMJD1 (KDM3) family*: There are three proteins in the JMJD1 family, including JMJD1A (JDHM2A, KDM3A), JMJD1B (JDHM2B, KDM3B) as well as JMJD1C (JDHM2C), all of which can demethylate H3K9me1/me2 (Fig. 5).^204,205^ It has been reported that JMJD1A can upregulate DCLK1 and CDK6 in a demethylation-dependent manner and maintain the occurrence and development of pancreatic cancer.^206,207^

*The JMJD2 (KDM4) family*: The JMJD2 (KDM4) family consists of six proteins (JMJD2A-F),^208,209^ all the proteins have the ability to demethylate H3K9me2/me3 and H3K36me2/me3, which are conductive to breast, colorectal, prostate and other cancers formation (Fig. 5).^210^ Studies have shown that overexpression of KDM4 proteins can change the transcription and chromatin remodeling to induce cell proliferation.^211^ However, knockout of KDM4 demethylases can reduce the expression of Taf1b and Nom1 genes by increasing the accumulation of H3K9me3 on the initiation site, so as to downregulate the maintenance of hematopoietic stem cells.^212^

*The JARID1 (KDM5) family*: There are four proteins named JARID1A, JARID1B, JARID1C, and JARID1D in the JARID1 (KDM5) family, which can demethylate methyl marks of H3K4.^213,214^ The structure of KDM5A contains three PHD domains, especially, the PHD1 domain in KDM5A can preferentially recognize unmethylated H3K4 histone tails and stimulate its activity (Fig. 5).^215^ It is worth noting that KDM5B is up-regulated in breast cancer and prostate cancer. Knocking down KDM5B can activate AMPK signaling pathway, through this manner, KDM5B reverses epithelial-mesenchymal transition (EMT), induces lipid reprogramming, and inhibits cell proliferation and migration, while its overexpression can enhance PI3K/AKT signal pathway.^216,217^ In clear cell renal cell carcinoma (ccRCC), JARID1C can significantly inhibit tumor growth by reducing H3K4me3 level.^218^ But JARID1C can regulate BRMS1 and its silence inhibits invasion and metastasis of breast cancer.^219^ Interestingly, as a transcription factor, KDM5D can demethylate Z2F1 to inhibit its expression, then inhibit its binding to FKBP4 and decrease the transcription of FKBP4.^220^

*The UTX/JMJD3 (KDM6) family*: In this family, UTX, also known as KDM6A and JMJD3 (KDM6B) discovered in 2007 can demethylate H3K27me2/me3,^221^ while UTY (KDM6C) has no enzymatic activity (Fig. 5).^222^ An increasing number of studies have indicated that JMJD3 plays an important role in maintaining the function of stem cells. JMJD3 can enhance neural commitment by regulating Pax6, Nestin, and Sox1 in order to influence the differentiation of Embryonic Stem Cell (ESC).^223^ In multiple myeloma, NF-κB pathway can upregulate KDM6B, and overexpression of KDM6B increases the expression of PRKCB and FOS genes related to MAPK pathway, thereby promoting the growth and survival of multiple myeloma cells.^224^ Notably, UTX is closely related to bladder cancer by affecting p53 and FGFR3 expressions.^225,226^

*The KDM7 family*: In KDM7 family, it is generally believed that KIAA1718 (KDM7A) and PHF8 (KDM7B) can participate in demethylating H3K27me1/2, H3K9me1/2 and H4K20me1 and promoting cancer progress (Fig. 5), while PHF2 is considered to inhibit tumor growth.^227,228^ Specifically, PHF8 is generally elevated in hepatocellular carcinoma (HCC), which is directly related to the occurrence and migration.^229^ Besides, PHF2 can modulate the expression of cell cycle-related genes and regulate DNA replication.^230^

In addition, there are many other proteins containing jumonji domain, for instance, similar to JARID1 family, although JARID2 does not have demethylase activity, its expression inhibits the formation of differentiation markers, and regulates keratinocyte differentiation genes.^231^ Besides, Hspbap1 containing jumonji C domain (jmjd) can interact with heat shock protein HSPb1, but its enzymatic activity needs further study and it only has the possibility of becoming a histone demethylase.^232^

##### Histone arginine demethylases

Like lysine methylation, histone arginine methylation is also a reversible process, but compared with demethylases for histone lysine, only a few histone arginine demethylases have been reported. So far, only two histone arginine demethylases have been reported.^233^ Peptidylarginine deiminase 4 (PAD4) can regulate arginine methylation and gene expression by removing methyl groups from H3R2, H3R8, H3R17, and H3R26, and then converting arginine to citrulline.^233,234^ When PAD4 activity is decreased, the expression of mesenchymal markers is increased, causing damages to cancer growth and metastasis.^235^ At the same time, knocking down PAD4 could promote cell autophagy and induce cell apoptosis, thereby inhibiting cell proliferation.^236^ JMJD6, another JmjC-containing iron and 2-oxoglutarate-dependent dioxygenase, can demethylate histone H3 arginine 2 (H3R2me2) and histone H4 arginine 3 (H4R3me2) as an arginine demethylase by removing methyl groups.^233,237,238^ Furthermore, JMJD1B can regulate the demethylation of H3K9me2, and then regulate the demethylation of H4R3me2/me1, which is closely related to the growth of hematopoietic stem cells (Fig. 5).^239^

#### Histone demethylase inhibitors in clinical development

**Fig. 7:**
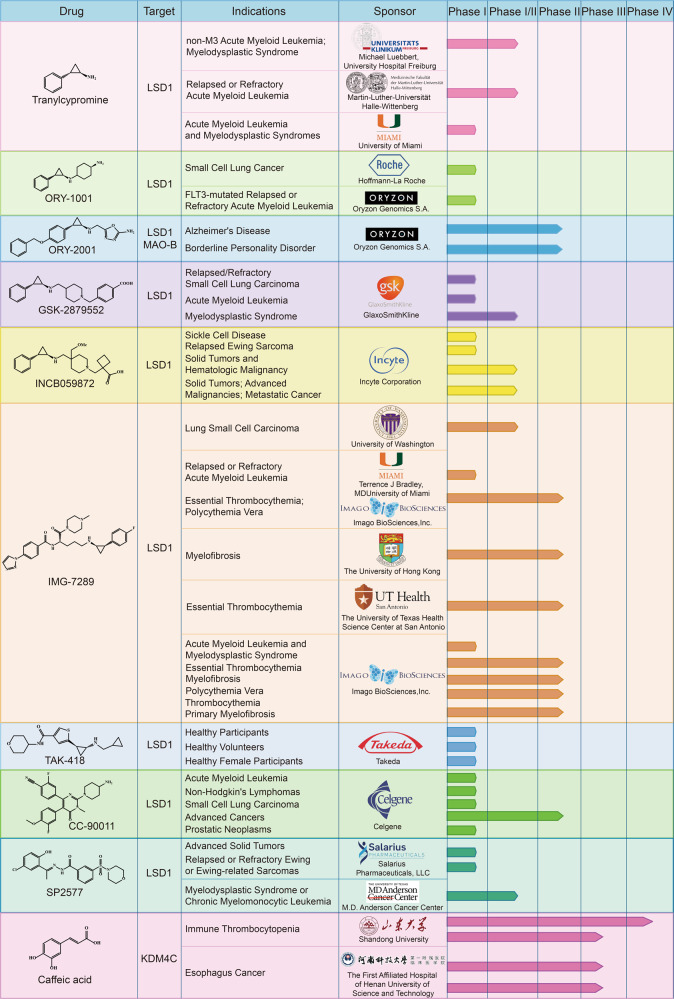
Representative inhibitors against histone demethylases in clinical trials

Given the importance of demethylases in tumorigenesis, the development of small-molecule inhibitors has become a central theme for cancer treatment. At present, many histone demethylase inhibitors have been developed, and some of them have been approved for use in clinical trials to evaluate their efficacy and safety in patients (Fig. 7, Table 1).^240,241^

**Table 1:** Inhibitors against histone demethylases in clinical trials

Drugs	Phase	Trial number	Indications	Status
Tranylcypromine	Phase I/II	NCT02717884	non-M3 Acute Myeloid Leukemia; Myelodysplastic Syndrome	Unknown
	Phase I/II	NCT02261779	Relapsed or Refractory Acute Myeloid Leukemia	Unknown
	Phase I	NCT02273102	Acute Myeloid Leukemia and Myelodysplastic Syndromes	Completed
ORY1001	Phase I	NCT02913443	Small Cell Lung Cancer	Completed
	Phase I	NCT05546580	FLT3-mutated Relapsed or Refractory Acute Myeloid Leukemia	Not yet recruiting
ORY-2001	Phase II	NCT03867253	Alzheimer’s Disease	Completed
	Phase II	NCT04932291	Borderline Personality Disorder	Recruiting
GSK2879552	Phase I	NCT02034123	Relapsed/Refractory Small Cell Lung Carcinoma	Terminated
	Phase I	NCT02177812	Acute Myeloid Leukemia	Terminated
	Phase I/II	NCT02929498	Myelodysplastic Syndrome	Terminated
INCB059872	Phase I	NCT03132324	Sickle Cell Disease	Terminated
	Phase I	NCT03514407	Relapsed Ewing Sarcoma	Terminated
	Phase I/II	NCT02712905	Solid Tumors and Hematologic Malignancy	Terminated
	Phase I/II	NCT02959437	Solid Tumors; Advanced Malignancies; Metastatic Cancer	Terminated
IMG-7289	Phase II	NCT05223920	Thrombocythemia Primary Myelofibrosis	Enrolling by invitation
	Phase I	NCT02842827	Acute Myeloid Leukemia and Myelodysplastic Syndrome	Completed
	Phase I	NCT05597306	Relapsed or Refractory Acute Myeloid Leukemia	Recruiting
	Phase I/II	NCT05191797	Lung Small Cell Carcinoma	Recruiting
	Phase II	NCT04254978	Essential Thrombocythemia	Active, not recruiting
	Phase II	NCT04262141	Essential Thrombocythemia; Polycythemia Vera	Recruiting
	Phase II	NCT03136185	Myelofibrosis	Completed
	Phase II	NCT05569538	Myelofibrosis	Recruiting
	Phase II	NCT04081220	Essential Thrombocythemia	Recruiting
	Phase II	NCT05558696	Polycythemia Vera	Not yet recruiting
TAK-418	Phase I	NCT03228433	Healthy Participants	Completed
	Phase I	NCT04202497	Healthy Volunteers	Terminated
	Phase I	NCT03501069	Healthy Female Participants	Terminated
CC-90011	Phase I	NCT04748848	Acute Myeloid Leukemia	Completed
	Phase I	NCT02875223	Non-Hodgkin’s Lymphomas	Active, not recruiting
	Phase I	NCT03850067	Small Cell Lung Carcinoma	Active, not recruiting
	Phase II	NCT04350463	Advanced Cancers	Active, not recruiting
	Phase I	NCT04628988	Prostatic Neoplasms	Recruiting
SP-2577	Phase I	NCT03895684	Advanced Solid Tumors	Completed
	Phase I	NCT03600649	Relapsed or Refractory Ewing or Ewing-related Sarcomas	Recruiting
	Phase I/II	NCT04734990	Myelodysplastic Syndrome or Chronic Myelomonocytic Leukemia	Recruiting
Caffeic acid	Phase III	NCT04648917	Esophagus Cancer	Unknown
	Phase III	NCT03070262	Esophagus Cancer	Unknown
	Phase IV	NCT02556814	Immune Thrombocytopenia	Completed
	Phase III	NCT02351622	Immune Thrombocytopenia	Completed

Originally developed as a MAO inhibitor to treat mood and anxiety disorders, tranylcypromine (TCP) can inhibit the activity of MAO-A and MAO-B.^242,243^ In addition, as an irreversible LSD1 inhibitor, TCP also inhibits cancer proliferation and invasion.^244^ A clinical phase I/II study investigating whether TCP could sensitize ATRA in patients with non-M3 AML or MDS in 2015 was initiated by Michael Luebbert and the University Hospital Freiburg (ClinicalTrials.gov Identifier: NCT02717884). In phase I trial, four dose levels of TCP (20 mg, 40 mg, 60 mg, 80 mg on days 1–28) were examined in combination with fixed dose of ATRA (45 mg/m^2^ on days 10–28) and fixed-dose of AraC (40 mg on days 1–10) to determine the dose used in phase II clinical trial, and then phase II trial was designed to evaluate the efficacy of TCP at recommended dose in patients with AML or MDS. In addition, two other clinical trials have been carried out for the combination of TCP and ATRA (ClinicalTrials.gov Identifier: NCT02261779; NCT02273102). Phase I/II clinical trials initiated by Martin-Luther-Universität Halle-Wittenberg was designed to explore the feasibility, safety, and efficacy of combining TCP with all-trans retinoic acid (ATRA) in relapsed or refractory AML.^245^ And another phase I clinical trial sponsored by University of Miami has been completed in 2020, it indicated that these two drugs combination was safe and effective, and LSD1 inhibition can sensitize AML cells to ATRA.

ORY-1001 is a highly potent and selective covalent LSD1 inhibitor.^246^ A phase I study on the safety, pharmacodynamics, and pharmacokinetics of ORY-1001 in relapsed or refractory AML sponsored by Oryzon Genomics has been successfully completed in 2016 (EudraCT 2013-002447-29). A phase I clinical trial to determine the maximum tolerated dose in participants with relapsed, extensive-stage disease small cell lung cancer has also been completed (ClinicalTrials.gov Identifier: NCT02913443), Moreover, the combination of ORY-1001 with driamycin, an inhibitor of DNA methylation, also entered phase II clinical trials (EudraCT 2018-000482-36). Very recently, a phase I study was sponsored by Oryzon Genomics S.A. to investigate the safety of the combination of ORY-1001 with gilteritinib in FLT3-mutated R/R AML (ClinicalTrials.gov Identifier: NCT05546580). ORY-2001 (vafidemstat) is another LSD1/MAO-B dual inhibitor developed by Oryzon Genomics.^239^ However, its clinical studies were mainly designed for treating Alzheimer’s disease (EudraCT 2019-001436-54; ClinicalTrials.gov Identifier: NCT03867253), acute respiratory distress syndrome (ARDS) (EudraCT 2020-001618-39), multiple sclerosis (MS) (EudraCT 2017-002838-23), borderline personality disorder (ClinicalTrials.gov Identifier: NCT04932291), etc, rather than tumors. GSK2879552 is a selective irreversible inhibitor developed by GlaxoSmithKline.^247^ Clinical trials evaluating its safety, pharmacokinetics and pharmacodynamics in recurrent or refractory SCLC (ClinicalTrials.gov Identifier: NCT02034123), AML (ClinicalTrials.gov Identifier: NCT02177812) and MDS (ClinicalTrials.gov Identifier: NCT02929498) have been terminated because of their poor risk benefit.

INCB059872 is a novel LSD1 inhibitor,^239^ and a phase I/II clinical study was started to evaluate its safety, tolerability and efficacy in patients with advanced malignant tumors in 2016, which was conducted in 4 parts (ClinicalTrials.gov Identifier: NCT02712905). Part 1 determined the recommended dose of INCB059872 based on maximum tolerated dose. Part 2 further determined the safety, tolerability, efficacy, PK, and PD of the selected monotherapy dose in various malignant tumors, including AML/MDS, SCLC, myelofibrosis, ewing sarcoma, and poorly differentiated neuroendocrine tumors. Part 3 determined the recommended dose of INCB059872 in combination with azacitadine and ATRA in AML and in combination with nivolumab in SCLC. And part 4 further determined the safety, tolerability, efficacy, PK, and PD of the selected combination dose in Part 3. However, this study has been terminated according to strategic business decision. In addition, clinical trials evaluating the safety and activity of INCB059872 in subjects with sickle cell disease (ClinicalTrials.gov Identifier: NCT03132324) and relapsed or refractory Ewing sarcoma (ClinicalTrials.gov Identifier: NCT03514407) have also been discontinued according to business decisions. A phase I/II study in subjects with advanced or metastatic solid tumors to evaluate the combination of INCB059872 with programmed death receptor-1 (PD-1) inhibitor pembrolizumab and the indoleamine 2,3-dioxygenase (IDO-1) inhibitor epacadostat has also been terminated by sponsor (ClinicalTrials.gov Identifier: NCT02959437).

As a selective LSD1 inhibitor developed by Imago BioSciences, there are ten studies that have been registered in *clinicaltrials.gov* website under IMG-7289. Very recently, a phase I clinical trial was started to evaluate the safety and tolerability of IMG-7289 and Bomedemstat combination therapy in relapsed or refractory acute myeloid leukemia (ClinicalTrials.gov Identifier: NCT05597306). A phase I clinical trial sponsored by Imago BioSciences to evaluate the safety, steady-state pharmacokinetics, and pharmacodynamics of IMG-7289 alone or in combination with ATRA in the treatment of AML and MDS patients has been completed (ClinicalTrials.gov Identifier: NCT02842827). In addition, a phase I/II study initiated by the University of Washington to explore the combination of IMG-7289 with the PD-L1 inhibitor atezolizumab in patients with small cell lung cancer is also recruiting volunteers (ClinicalTrials.gov Identifier: NCT05191797). Subjects involved in the study received atezolizumab intravenously on day 1 and IMG-7289 once daily on days 1-21, and cycles were repeated every 21 days in the absence of disease progression or unacceptable toxicity. Then these patients were followed up at 30 days and every 12 weeks thereafter after treatment completion. Moreover, Imago BioSciences also launched a phase II clinical study in 2021 to evaluate the safety and effectiveness of IMG-7289 in patients with myopathic neuroplasms (ClinicalTrials.gov Identifier: NCT05223920). And the remaining clinical studies were to evaluate the effect of IMG-7289 on the essential thrombocythemia (ClinicalTrials.gov Identifier: NCT04254978; NCT04262141; NCT04081220), Polycythemia Vera (ClinicalTrials.gov Identifier: NCT05558696) and Myelofibrosis (ClinicalTrials.gov Identifier: NCT03136185; NCT05569538). Another LSD1 small-molecule inhibitor TAK-418,^239^ a phase I study initiated by Takeda to evaluate its safety and tolerability when administered as a single oral dose in healthy participants has been completed (ClinicalTrials.gov Identifier: NCT03228433). The participants were divided into five groups, six of them received TAK-418 and two received matching placebo. The results showed that TAK-418 was well tolerated and exhibited a nearly linear pharmacokinetic profile with a *t*_1/2_ of 4.35–5.36 h.^248^ Moreover, other two phase I studies in healthy volunteers after oral dose of TAK-418 administration have been terminated because of administrative reasons and business decision, respectively (ClinicalTrials.gov Identifier: NCT04202497, NCT03501069).

In addition to covalent irreversible LSD1 inhibitors, two LSD1 reversible inhibitors have also entered clinical trials.^249,250^ Most clinical trials of the first reversible LSD1 inhibitor CC-90011 are related to drug combinations. For example, a phase I/II study was supported by Celgene to assess the safety, tolerability, and preliminary efficacy of CC-90011 given concurrently with venetoclax and azacytidine in AML, this clinical trial was completed in 2022 (ClinicalTrials.gov Identifier: NCT04748848). It included three parts: a dose escalation part in R/R AML, a dose escalation part in newly diagnosed AML (ndAML), and a randomized dose expansion part in ndAML of Venetoclax and Azacitidine with or without CC-90011. Another phase I study aimed to evaluate the safety and efficacy of CC-90011 in combination with itraconazole and rifampicin in patients with relapsed and refractory solid tumors and non-Hodgkin’s lymphomas was initiated in 2016 (ClinicalTrials.gov Identifier: NCT02875223). The study comprised two parts, the dose escalation part (part A) aimed to estimate the maximum tolerated dose (MTD) of CC-90011, and the expansion part (part B) further evaluated the safety and efficacy of CC-90011 administered at or below the MTD in 3 selected expansion cohorts in order to define the recommended phase 2 dose (RP2D). In addition, a phase I trial of CC-90011 in combination with cisplatin and etoposide (ClinicalTrials.gov Identifier: NCT03850067) and a phase II trial in combination with nivolumab (ClinicalTrials.gov Identifier: NCT04350463) in SCLC has also been initiated. In addition to the drug combination, a phase I study aimed to assess whether CC-90011 can induce AR expression to re-sensitize metastatic castration-resistant prostate cancer to anti-hormonal therapy is recruiting (ClinicalTrials.gov Identifier: NCT04628988). SP-2577 is also a reversible LSD1 inhibitor developed by Salarius Pharmaceuticals. A phase I clinical study of SP-2577 in patients with advanced solid tumors has been completed (clinicalTrials.gov Identifier: NCT03895684). In this study, SP-2577 was given as oral tablets in patients with advanced solid tumors with a 28-day cycle. And clinical trials of SP-2577 in combination with topotecan and cyclophosphamide (ClinicalTrials.gov Identifier: NCT03600649), as well as azacytidine (ClinicalTrials.gov Identifier: NCT04734990), have also been initiated.

Compared with LSD1 inhibitors, very few inhibitors targeting other demethylases have entered clinical trials. Coffeic acid inhibits KDM4C expression.^251^ Two phase III studies were initiated by the First Affiliated Hospital of Henan University of Science and Technology to investigate the efficacy and safety of coffeic acid in Chinese advanced esophageal squamous cell cancer (ClinicalTrials.gov Identifier: NCT04648917, NCT03070262). In addition, a phase III clinical trial, a multicenter randomized study of caffeic acid tablets as second-line therapy for the treatment of immune thrombocytopenia (ITP), was initiated by Shandong University in 2012 (ClinicalTrials.gov Identifier: NCT02351622). Then a phase IV trial was also initiated by Shandong University to investigate the efficacy and safety of caffeic acid tablets combined with dexamethasone in ITP (ClinicalTrials.gov Identifier: NCT02556814).

As an emerging target for cancer therapy, LSD1 has important biological roles in multiple biological processes and diseases. Until now, many LSD1 inhibitors have been reported, including reversible and irreversible inhibitors. TCP has been recognized as a privileged scaffold for new irreversible LSD1 inhibitors and six TCP-based irreversible inhibitors alone or in combination with other therapeutic agents have been in clinical trials for disease treatment (Table 1). However, these LSD1 irreversible inhibitors can be covalently conjugated to FAD, resulting in a long-term inhibitory effect on the FAD-dependent targets, therefore, it may increase the off-target effect and potential toxicity.^241^ For example, the phase I clinical trial initiated by University of Miami showed that common adverse effects of TCP are febrile neutropenia and increased creatinine (ClinicalTrials.gov Identifier: NCT02273102). And all three clinical trials of GSK2879552 were terminated due to its potential toxic effects, poor disease control, and poor risk benefit. In contrast, reversible inhibitors have safer profiles, so developing highly active and selective reversible LSD1 inhibitors is a central focus currently. The discovery of CC-90011 and SP-2577 proves the therapeutic potential of reversible inhibitors (Table 1). Notably, LSD1 has interactions with other proteins, and combination therapy of LSD1 inhibitors and other drugs may have synergistic effects. Currently, all the combinations of TCP with ATRA, ORY-1001 with gilteritinib, INCB059872 with azacitadine and ATRA, IMG-7289 with bomedemstat, and atezolizumab, CC-90011 with venetoclax and azacytidine, SP-2577 with topotecan and cyclophosphamide, as well as azacytidine have initiated clinical trials, demonstrating the importance of drug combinations. Unfortunately, compared to LSD1, few other demethylases inhibitors have entered clinical trials.

#### Histone demethylases and cancer drug resistance

Epigenetic changes, especially histone methylation, and demethylation, play an important role in drug resistance.^252^ As regulatory factors, histone demethylases are closely related to drug resistance, and targeting the demethylases in various tumors represents a promising strategy to overcome drug resistance (Fig. 3). In this section, we mainly discussed the functions and effects of different histone demethylases on drug resistance.

##### The lysine-specific demethylase (LSD) family and drug resistance

LSD1 is an important histone demethylation enzyme and can regulate the drug resistance of various cancer cells by changing the methylation levels of H3K4 and H3K9.^181,182^ Satoi Nagasawa et al. found that the increased LSD1 mRNA level is a potential prognostic factor of poor prognosis in basal-like breast cancer, and the increased expression of LSD1 protein is related to the poor prognosis of triple-negative breast cancer.^253^

**Fig. 8:**
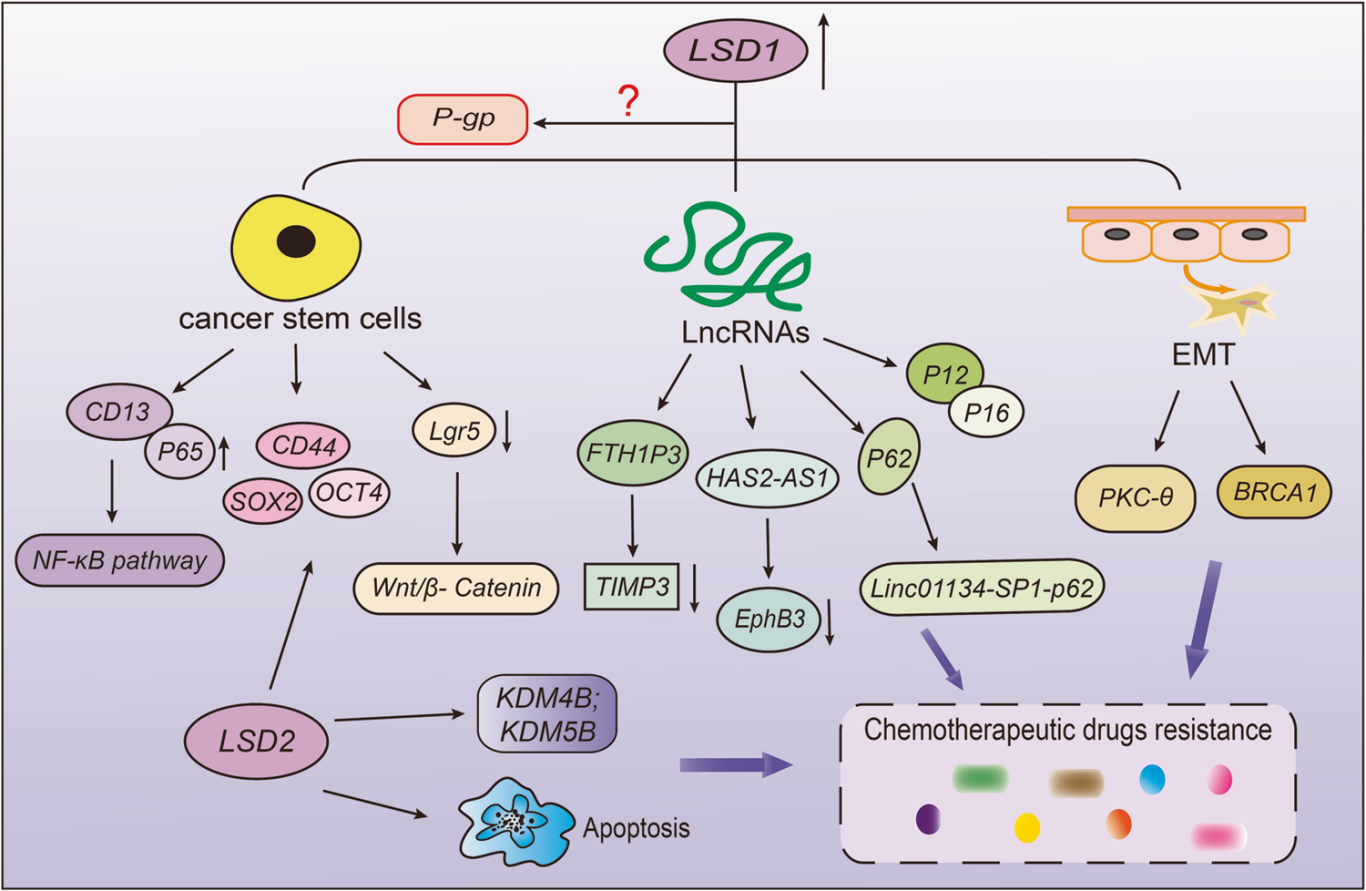
The roles of lysine-specific demethylase (LSD) family on drug resistance. LSD1 can maintain the function of stem cells by activating NF-κB and Wnt/β-catenin signaling pathway, promote EMT and interact with long-chain non-encoding RNA, thus promoting drug resistance. LSD2 can enhance stem cell characteristics, induce cell apoptosis, and regulate other enzyme expressions to promote drug resistance

It is generally acknowledged that cancer stem cells have a strong ability for self-renewal, differentiation, and proliferation.^254–256^ Therefore, conventional chemotherapy cannot completely eliminate CSCs in cancer cells, and there remain a lot of residues of tumor stem cells after chemotherapy, which is an important reason for drug resistance.^257,258^ As a critical epigenetic enzyme, LSD1 can further regulate cell resistance by modulating the function of stem cells (Fig. 8). As reported, John Verigos et al. found that overexpression of LSD1 increased the ability of mammary gland formation and the stem cell potential, thereby increasing the resistance of breast cancer cells to adriamycin, while knocking down LSD1 led to the opposite effect.^259^ In this sense, the high level of LSD1 is associated with poor prognosis in breast cancer patients (Table 2). In addition to breast cancer, LSD1 can also regulate drug resistance in many other cancers by changing cell viability, such as colorectal cancer, gastric cancer, and hepatocellular carcinoma. Tumor stem cells have many surface markers, such as CD13, CD133 and CD44.^260,261^ Hu et al. have demonstrated that CD13 can prevent LSD1 from protein ubiquitination and degradation to stabilize LSD1 via deacetylating LSD1 by HDAC5. Next, LSD1 enhanced the demethylation level of p65 protein to improve the stability of p65 and activate NF-κB consequently, eventually producing sorafenib resistance.^262^ Based on this, knocking down LSD1 can be used as an important target for the treatment of colorectal cancer by attenuating CD133^+^ cell stemness characteristics (Table 2).^263^ In addition, gastric cancer cells can release small extracellular vesicles containing LSD1, which can increase the expression of CD44, SOX2, and OCT4, thus promoting chemoresistance to oxaliplatin.^264^ Significantly, it also indicated that LSD1 plays an important role in cancer stem cells after long-term sorafenib therapy in HCC (Table 2).^265^ Decreasing LSD1 can inhibit Wnt/β-catenin signaling pathway to enhance the sensitivity of drug-resistant cells to sorafenib.^265^ And a recent study showed that LSD1 increased cancer stemness by activating Wnt/β-catenin signaling pathway, inducing thyroid cancer resistance to doxorubicin.^266^ At the same time, LSD1 can inhibit the expression of some suppressors, especially spirgle1 and APC in Lgr5+ cancer-initiating cells by regulating H3K4me1/2 methylation, resulting in high viability of Lgr5+ cells and occurrence of cancer cell drug resistance.^267^

**Table 2:** Effect of LSD family on drug resistance

Enzyme	Cancer type	Drug	Effect or mechanism	Reference
LSD1	breast cancer	adriamycin	increase the ability of mammary gland formation and cancer stem cell potential	^259^
	hepatocellular carcinoma	sorafenib	improve the stability of p65 protein, and activate NF-κB consequently; inhibit the expression of spirgle1 and APC to maintain the viability of Lgr5+ cells	^262,265^
	gastric cancer	oxaliplatin	increase the expression of CD44, SOX2 and OCT4, and promote chemoresistance	^264^
	thyroid cancer	doxorubicin	increase cancer stemness by activating Wnt/β-catenin signaling pathway	^266^
	NSCLC	gefitinib	bind with FTH1P3 and TIMP3 promoter region to strengthen cell resistance to gefitinib	^269^
	hepatocellular carcinoma	oxaliplatin	activate the LINC01134-SP1-p62 axis	^271^
LSD2	breast cancer	DAC (a DNMT inhibitor)	knocking down LSD2 can inhibit cell clonogenic ability, induce cell apoptosis and block cells in S phase	^278^
	ovarian cancer	cisplatin	inhibition of LSD2 decrease the level of DNA damage repair gene DCLRE1B	^280^

Long chain non-encoding RNA is a transcription factor consisting of >200 nucleotides, and it lacks protein encoding potential. LncRNAs can participate in regulating drug resistance and multiple cellular processes through changing epigenetic and gene expressions in translation level (Table 2).^268^ Many studies have shown that LncRNAs can interact with and recruit LSD1 in order to regulate cell resistance through demethylation modification (Table 2, Fig. 8).^269–272^ Zheng et al. found that the poor prognosis in NSCLC patients was closely related to overexpression of LncRNA FTH1P3, which can inhibit TIMP3 protein by recruiting LSD1 and increased cell resistance to gefitinib.^269^ Moreover, HAS2-AS1 also inhibited EphB3 by recruiting LSD1, promoting tumorigenesis and gefitinib-resistance in NSCLC.^270^ Li et al. have found that in gefitinib-resistance cell PC-9, the expression of Hox transcript antisense intergenic RNA (HOTAIR) was up-regulated, while the level of LSD1 decreased significantly, which increased the recruitment of H3K27me3 to p16 and p21 promoters.^272^ In oxaliplatin resistant HepG2 cells, the high expressions of LINC01134, p62 and LSD1 were positively correlated. LSD1 can activate the expression of LINC01134 and increase the resistance of HCC to oxaliplatin through the LINC01134-SP1-p62 axis.^271^ In summary, LncRNAs can interact with LSD1 and regulate the generation of drug resistance collectively.

In addition to the above mechanisms, LSD1 has also been reported to promote drug resistance in breast cancer and NSCLC by regulating EMT (Fig. 8).^273,274^ LSD1 is essential for hypoxia-induced gefitinib-resistance, and knockout LSD1 can reverse resistance and enhance drug sensitivity by blocking the associated EMT.^273^ Boulding et al. have proved for the first time that LSD1 can interact with PKC-θ during the EMT process in breast cancer and promote gene induction.^274^ Importantly, silencing LSD1 can also increase the BRCA1 mRNA level in breast cancer cells (Table 2).^275^ Taken together, LSD1 can induce cell resistance through various mechanisms, such as maintaining the function of cancer stem cells, regulating EMT process and interacting with small interfering RNA. Interestingly, although it has been proved that multidrug resistance genes and ABC binding proteins are important factors in the development of drug resistance, the clear relationship between LSD1 and them, especially the P-glycoprotein, has not yet been elucidated.^6,276^ Therefore, more attention might need to be devoted to the effect of LSD1 on P-gp expression for overcoming drug-induced resistance.

Up to now, compared with LSD1, the functions of LSD2 on drug resistance are less studied. A recent study has indicated that LSD2 is highly expressed in PANC-1, and knocking down LSD2 can inhibit cell proliferation by increasing the apoptosis rate and the related apoptotic proteins expressions.^277^ Similarly, Tiffany et al. found that knocking down LSD2 can inhibit cell clonogenic ability, induce cell apoptosis, and block cells in S phase, thereby making breast cancer cell sensitive to 5-aza-deoxycytidine (DAC), a DNMT inhibitor (Table 2).^278^ Chen et al. proved for the first time that the overexpression of LSD2 increased NANOG, SOX2, and mRNA levels of LSD1, KDM4B and KDM5B at the same time, and promoted the characteristics of CSCs, suggesting that LSD2 might interact with other enzymes in tumor development.^279^ Moreover, LSD2 gene may be the key to the resistance to cisplatin in ovarian cancer. When LSD2 was inhibited, the expression of DNA damage repair gene DCLRE1B decreased, and the sensitivity of cells to cisplatin increased significantly, thus inhibiting cell growth.^280^

##### The JmjC domain-containing protein family and drug resistance

**Fig. 9:**
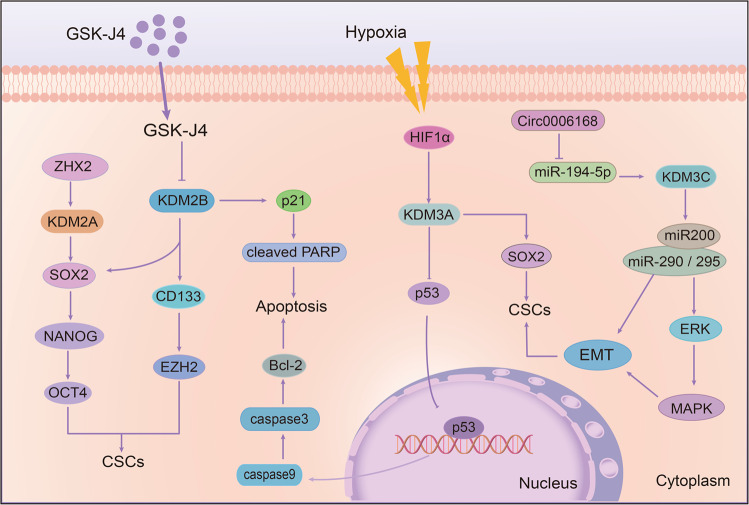
The roles of KDM2 and KDM3 families on drug resistance. KDM2A can promote cell stemness by upregulating the expression of stemness markers Sox2 and Oct4. KDM2B can enhance drug resistance by increasing stem cells and apoptosis at the same time. In addition, KDM3A is able to decrease p53 expression at the transcriptional level and KDM3C can inhibit EMT through the ERK/MAPK signal pathway

*KDM2 family and drug resistance*: There are two members in KDM2 family. Recent studies have shown that the KDM2 family can promote the emergence of cell resistance. As the target of Zinc-fingers and homeoboxes (ZHX2), a tumor suppressor, KDM2A is negatively correlated with ZHX2 in hepatoma cells. KDM2A knockdown can lessen its downstream SOX2, NANOG, and OCT4 to reduce the formation of tumor stem cells and decrease the resistance to sorafenib (Table 3).^281^ Another member of the KDM2 family, KDM2B, also plays a significant role in the regulation of stem cells, thereby regulating cell sensitivity to drugs. Staberg et al. reported that targeting KDM2B can reduce the expression of EZH2, CD133, and SOX2 and impair the differentiation ability of stem cells, which is necessary for glioblastoma (GBM). Moreover, the deletion of KDM2B also increased the apoptotic cells by inducing the expression of p21^CIP1/WAF1^ and cleaved PARP, thus making cells more sensitive to chemotherapeutic agents (Table 3).^282^ Besides, inhibition of KDM2B in GBM led to a higher expression of tumor necrosis factor-related apoptosis-inducing ligand (TRAIL) and reduced tumor growth and angiogenesis in mice (Fig. 9).^283^

**Table 3:** Effects of proteins containing jumonji domain on drug resistance

Enzyme	Cancer type	Drug	Effect or mechanism	Reference
KDM2A	hepatoma	Sorafenib	Increase the formation of tumor stem cells	^281^
KDM2B	glioblastoma	Chemotherapeutic agents	Induce the expression of p21^CIP1/WAF1^ and increase the apoptosis	^282^
KDM3A	breast cancer	Cisplatin	Activate SOX2 and inhibit p53 activity to regulate BCL-2 expression	^287^
JMJD1C	ESCC	Paclitaxel	Inhibit cell apoptosis rate and reduce metastasis	^290^
KDM4A	castrated resistant prostate cancer	Enzalutamide	Regulate the stability of USP14-AR and prevent AR degradation	^295^
KDM4B	prostate cancer	Enzalutamide	Increase the transcription of C-MyC	^297^
KDM4C	acute myeloid leukemia	Cytarabine	Increase the expression of MALAT1 and activate cyclin CCND2	^299^
	gastric cancer	Cisplatin	Activate ALDH1A3 transcription and maintain stemness	^301^
KDM5A	glioblastoma	Temozolomide	Promote resistance and inhibit apoptosis	^304^
	ER (+) breast tumor	Tamoxifen	Activate IGF1R and ErbB signaling, thereby causing the activation of PI3K/AKT/mTOR pathway	^305^
	lung adenocarcinoma	Paclitaxel	Increase the expression of P-gp; promote EMT	^307^
KDM5B	gastric cancer	Cisplatin	Recruit XRCC1 and inhibit cell apoptosis	^309^
KDM5C	CRPC; nasopharyngeal carcinoma; gastric cancer	Chemotherapeutic agents	Inhibit apoptosis and promote cell proliferation and resistance	^315,316^
	colorectal cancer; ccRCC	Oxaliplatin;	Promote cell growth while its knockdown promote resistance	^318,319^
KDM5D	prostate cancer	Docetaxel	Regulate androgen transcription; inhibit MYBL2 expression	^321,322^
KDM6A	AML	AraC; imatinib	Increase ENT1; upregulate TRKA expression through YY1	^325,326^
KDM6B	neuroblastoma	Palbociclib	Activate the CDK4/6-pRB-E2F pathway	^335^
KDM7A	bladder cancer	Cisplatin	Regulate AR transcription activity	^336^
PHF8	breast cancer	Trastuzumab	Interact with HER2 and regulate the expression of IL6	^338^
JARID2	non-small cell lung cancer	Cisplatin	Increase the expression of Notch1 and regulate cell stemness	^339^

*KDM3 family and drug resistance*: Several evidences have shown that KDM3A is closely related to radiation and drug resistance. Under hypoxia, KDM3A was increased and co-localized with hypoxia-inducible factor HIF-1α in esophageal squamous cell carcinoma (ESCC). When KDM3A was knocked down, the sensitivity of cells to radiation therapy can be increased, and the survival rate of cells under hypoxia conditions was significantly reduced.^284^ Notably, p53 is an important target of KDM3A, and KDM3A can inhibit the transcriptional activity of p53 by demethylating p53. On the contrary, when KDM3A was inhibited, the binding of p53 to PUMA and NOXA promoter was strengthened, and the expression of related apoptosis proteins was increased, thus restoring cell sensitivity.^285,286^ Moreover, Ramadoss et al. also found that in cisplatin-resistant ovarian cancer cells, KDM3A can activate SOX2 and inhibit p53 activity through demethylating p53, thereby downregulating the expression of apoptosis-related protein BCL-2 and p21, while inhibiting KDM3A can block the drug-resistant cells in G2/M phase (Fig. 9, Table 3).^287^ In addition to affecting p53, Wade et al. demonstrated that KDM3A is essential for the expression of ER-targeted genes in tamoxifen-resistant cells, and its deletion can inhibit cell proliferation by blocking cells in G1 phase.^288^ Overall, KDM3A plays a crucial role in drug resistance. It mainly affects the function of p53 by methylation modification, and then regulates downstream genes of p53 to promote drug resistance and cell proliferation.^285–287^ Therefore, targeting KDM3A could be a feasible strategy to overcome resistance.

Although studies on KDM3B and KDM3C are relatively rare, these homologs play a crucial role in promoting tumor growth and drug resistance. The function of JMJD1A and JMJD1B to demethylate H3K9 is necessary to maintain the function of embryonic stem cells (ESC). Their depletion increased the rate of apoptotic cells and weakened the versatile nature of ESC.^289^ Moreover, apart from H3K9me2, KDM3B can also demethylate H4R3me2, which affects the development and function of hematopoietic stem cells.^239^ Notably, the expression of JMJD1C increased significantly in paclitaxel-resistant ESCC. When JMJD1C was knocked down, the cell metastasis ability was reduced, and cell apoptosis rate was increased, so that it could reduce resistance (Table 3).^290^ Similarly, Schimek et al. also found that the sensitivity of cells to gemcitabine and carboplatin was increased after knocking down JMJD1C.^291^ Moreover, ESC self-renewing needs JMJD1C, which is likely to inhibit ERK/MAPK signal transduction and EMT process activation by regulating the miR-200 family and miR-290/295 cluster (Fig. 9).^292^

**Fig. 10:**
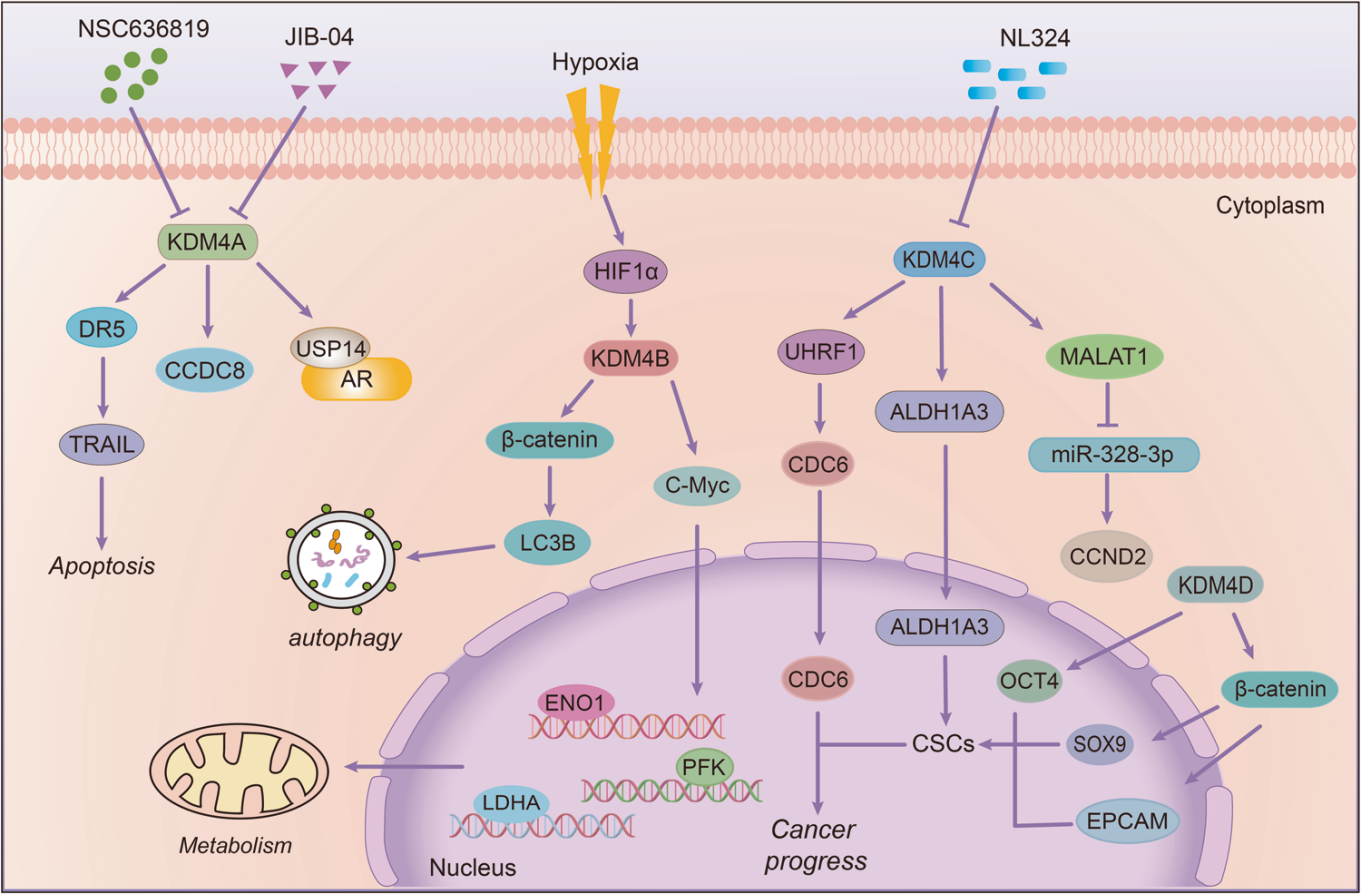
The role of KDM4 family in drug resistance. Small-molecule inhibitors NSC636819 and JIB-04 inhibit KDM4A activity and increase cell sensitivity by affecting CCDC8 expression and apoptosis. Under hypoxia, KDM4B can induce autophagy and increase metabolism through activating the Wnt/β-catenin pathway and transcribing C-Myc respectively. At the same time, KDM4C is able to promote cancer progress by influencing cell cycle and transcription of CDC6, and KDM4D can increase the CSC property through the Wnt/β-catenin and Notch signaling pathways

*KDM4 family and drug resistance*: There are six members of the KDM4 family. In addition to JMJD2E and JMJD2F, other four members are frequently overexpressed in cancer and closely involved in the development of drug resistance. Metzger et al. first demonstrated that KDM4A controlled the proliferation and self-renewal of breast cancer stem cells (BCSCs) which have been suggested to be responsible for therapy resistance.^293^ Besides, JMJD2A is tightly related to the sensitivity of gastric cancer cells to anticancer drugs, which can be regulated by the interaction between JMJD2A and its substrate CCDC8.^294^ In castrated resistant prostate cancer (CRPC), the expression of KDM4A-AS1 was increased, which could regulate the stability of USP14-AR and prevent AR degradation, and this was why cells were resistant to enzalutamide (Table 2).^295^ Apart from KDM4A, KDM4B also plays an important role in prostate cancer. Mechanically, HIF-1α induced expression of KDM4B to promote cell proliferation by activating the Wnt/β-catenin pathway and activating autophagy.^296^ It has been found that KDM4B can interact with C-Myc and promote metabolic genes LDHA, ENO1, and PFK levels to regulate cellular metabolism (Fig. 10).^297^ Furthermore, upon interacting with AR, it can also increase the transcription of C-Myc through its demethylation of H3K9, and its upregulation promotes cell resistance to enzyluamine (Table 3).^298^ Taken together, these results suggest that targeting KDM4B could be a potential strategy for treating CRPC.

Like KDM4A and KDM4B, high expression of KDM4C was also closely related to drug resistance. KDM4C can increase the expression of MALAT1 and MALAT1 inhibits its downstream miR-328-3p expression, and then activates cyclin CCND2, making the acute myeloid leukemia (AML) cells resistant to cytarabine (Fig. 10, Table 3).^299^ In addition, UHRF1 can recruit KDM4C and further regulate the transcription of CDC6 through the demethylation of H3K9 by KDM4C, which is crucial for cell resistance.^300^ Similar to the function of LSD1, KDM4C, and KDM4D are also necessary for the maintenance of cancer stem cell. Overexpression of KDM4C could activate aldehyde dehydrogenase ALDH1A3 transcription, promoting stemness and chemoresistance of gastric cancer stem cells.^301^ Chen et al. reported that KDM4C could bind to OCT4 promoter and affect its expression. When the expression of KDM4C was decreased, the migration and spheroid formation ability of cells was reduced, and the CSC property was inhibited.^302^ And it has been proved that JMJD2D can enhance the transcription of EpCAM and Sox9, which are the target genes of Wnt/β-catenin signaling pathway and Notch signaling pathway, thereby promoting the self-renewal ability of liver cancer stem-like cells (LSCS) (Fig. 10).^303^ Although the role of KDM4D on drug resistance is clear in hepatocellular carcinoma, the drug resistance mechanisms of KDM4D in other cancers are less studied. The roles of JMJD2E and JMJD2F on drug resistance are currently still unclear.

**Fig. 11:**
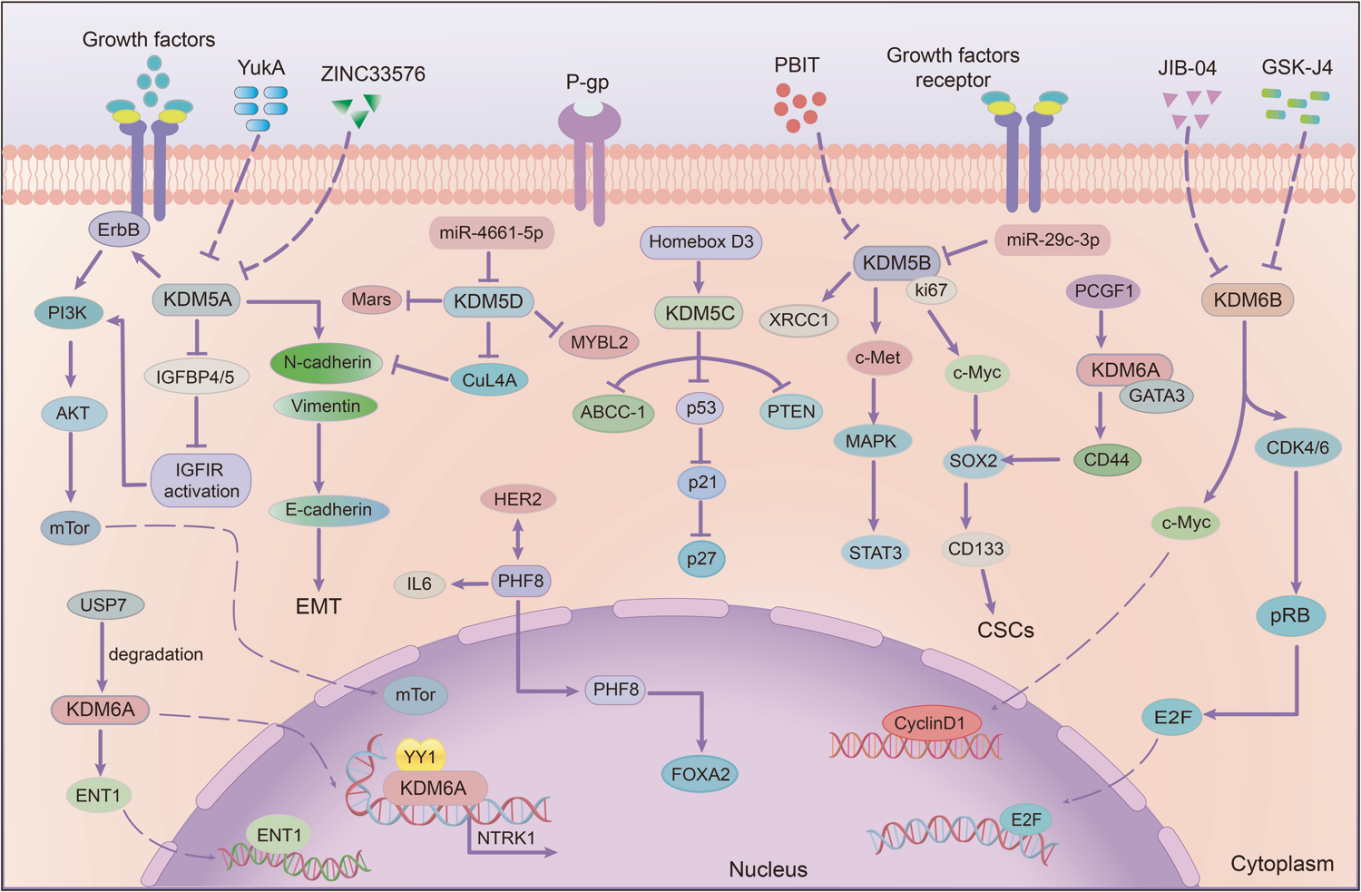
The roles of KDM5-7 on drug resistance. In KDM5 family, KDM5A can promote PI3K/AKT/mTOR pathway through ErbB and induce EMT in combination with KDM5D inhibition, thereby leading to drug resistance. However, KDM5C exerts opposite effects by regulating PTEN and p53, PBIT can inhibit the demethylase activity of KDM5B, which in turn affects the MAPK pathway and cell stemness. Besides, KDM6A and KDM6B can enhance drug resistance by promoting cell stemness and regulating the cell cycle, respectively. Importantly, PHF8 can also upregulate the expression of IL6 and FOXA2 to promote drug resistance

*KDM5 family and drug resistanc*e: The KDM5 family include JARID1A, JARID1B, JARID1C, and JARID1D, all of which have a significant impact on drug resistance. KDM5A is highly expressed in various cancers.^304–306^ In 2015, Romani et al. reported that in temozolomide-resistant glioblastoma cells, the expression of KDM5A was significantly elevated and its knockdown could promote cell apoptosis.^304^ In tamoxifen-resistant ER (+) breast tumors, KDM5A can activate IGF1R and ErbB signaling, causing the activation of PI3K/AKT/mTOR pathway and leading to the occurrence of tamoxifen resistance (Fig. 11, Table 3).^305^ What is more, inhibition of KDM5A can enhance the anti-proliferative ability of WEE1 inhibitor AZD1775 in drug-resistant acute myeloid leukemia cells.^306^ KDM5A also plays a key role in regulating P-gp and EMT. In detail, KDM5A can increase the expression of P-gp, inhibit cell apoptosis and promote cell proliferation (Table 3), at the same time, overexpression of KDM5A can also promote EMT) and metastasis of paclitaxel-resistant lung adenocarcinoma cells (Fig. 11).^307^

Like KDM5A, KDM5B is also highly expressed in many cancers and can promote drug resistance. In NSCLC, KDM5B can promote stem cell phenotypes by activating c-Met signaling pathway. While knockdown of KDM5B inhibits cell proliferation, migration, and colony-forming ability.^308^ Xu et al. demonstrated that KDM5B was highly expressed in cisplatin-resistant gastric cancer cells. It recruited XRCC1 through demethylation of H3K4, thus inhibiting cell apoptosis and increasing cisplatin resistance (Table 3).^309^ KDM5B is also highly expressed in paclitaxel-resistant endometrial cancer cells, which is regulated by microRNA-29c-3p (a tumor suppressor), thereby promoting drug resistance (Fig. 11).^310^ Importantly, KDM5B is also closely related to the drug resistance of melanoma, and the expression of KDM5B increased significantly after radiotherapy.^311^ KDM5B can induce the transformation of CD34 into CD34^+^ and decrease the sensitivity to BRAF inhibitors in mouse melanoma cells.^312^ What is more, it can be co-expressed with Ki67 and positively correlated with the proliferative ability of cells in canine tissues (Fig. 11).^313^

KDM5C is also a drug resistance regulator, but it has been recently reported to have two opposite effects. KDM5C can promote cell proliferation, but can restrain drug resistance. Hong et al. found that in CRPC, KDM5C can promote cell proliferation by inhibiting tumor suppressor PTEN regulated by BRD4 in vivo and in vitro (Fig. 11).^314^ And overexpression of KDM5C can promote nasopharyngeal carcinoma (NPC) cell proliferation and inhibit its apoptosis, while KDM5C knockdown exhibited the opposite results.^315^ KDM5C is highly expressed in gastric cancer cells and can promote cell proliferation and migration by inhibiting the expression of p53 and its downstream p21 and p27.^316^ And the latest study indicates that Homebox D3 can increase the expression of KDM5C and then inhibit p53, thereby promoting the proliferation of diffuse-large B-cell lymphoma (DLBCL).^317^ But interestingly, Lin et al. have demonstrated that KDM5C overexpression can reduce the expression of ABCC1 through its demethylation, thus inhibiting the resistance of colorectal cancer cells to drugs, such as oxaliplatin and irinotecan (Fig. 11).^318^ At the same time, knockdown of KDM5C can promote tumor growth and the resistance of ccRCC to ferroptosis (Table 3).^319^ Collectively, this evidence indicated that KDM5C might play different roles in different cancer cells. Compared with other KDM5s, the roles of KDM5D on drug resistance are less investigated. However, contrary to other members in KDM5 family, the decreased expression of KDM5D is beneficial to the drug resistance of cancer cells. It has been reported that low expression of KDM5D can promote the metastasis of gastric cancer cells by inducing EMT and demethylating H3K4 on CUL4A promoter (Fig. 11).^320^ Besides, Komura et al. indicated that the deletion or decrease of KDM5D is the major cause of docetaxel resistance. KDM5D can directly regulate the transcriptional activity of androgen, thereby affecting the sensitivity of CRPC to docetaxel.^321^ Additionally, silencing KDM5D could elevate MYBL2, thereby inducing docetaxel resistance in prostate cancer cells.^322^ As reported, miR-4661-5p is the upstream regulator of KDM5D. When it is inhibited, the expression of KDM5D is increased, and the expression of Mars2 is decreased, leading to a decrease in malignant behavior of cells (Fig. 11).^323^ Collectively, the KDM5 family are critical in the drug resistance, and targeting the KDM5 family is an important way to improve the curative effect.

*KDM6 family and drug resistance*: The KDM6 family plays an important role in drug resistance and cancer recurrence by demethylation of H3K27. Amongst them, KDM6A is a novel relapse-associated gene in AML.^324^ The reduction of KDM6A expression may decrease ENT1 level and is associated with a decreased cytarabine sensitivity.^325^ Besides, KDM6A can upregulate TRKA expression through YY1 independent of its demethylation activity, thereby promoting cell resistance to imatinib (Table 3).^326^ In addition to leukemia, KDM6A is also a key regulator in osteosarcoma, colorectal cancer, and prostate cancer. One study suggested that KDM6A was up-regulated in prostate cancer. USP7 can increase KDM6A expression by deubiquitinating it, while targeting KDM6A can inhibit tumor growth (Fig. 11).^327^ Furthermore, the low level of H3K27me3 is closely related to cell stemness and oxaliplatin resistance in colorectal cancer. PCGF1 can increase the expression of KDM6A, further reducing H3K27me3 level to activate the transcription of stem cell markers and promoting the proliferation of stem cells.^328,329^ What is more, the transcription factor GATA3, which is closely related to the poor prognosis of ovarian cancer, can recruit UTX and enhance the stemness of ovarian HGSC cells.^330^

Like KDM6A, KDM6B is highly expressed in various cancers and is conducive to tumor development and drug resistance. In DLBCL, inhibition of KDM6B can significantly increase the sensitivity of cells to chemotherapeutic drugs.^331^ Besides, KDM6B increased the expression of IGFBP5, and its inhibition can induce apoptosis and increase the sensitivity of breast cancer cells to GDC-0941.^332^ Notably, KDM6B can facilitate the expression of C-Myc and its target gene CyclinD1, thereby promoting the proliferation of prostate cancer cells and tumor growth.^333,334^ Interestingly, KDM6B is overexpressed in neuroblastoma and can activate the CDK4/6-pRB-E2F pathway, which is closely related to palbociclib resistance by demethylation of H3K27 (Fig. 11, Table 3).^335^ These data suggest that inhibition of KDM6B could become an effective therapeutic strategy to overcome drug resistance.

*KDM7 family and drug resistance*: Until now, the roles of the KDM7 family on drug resistance have less been studied. KDM7A can upregulate AR transcription activity *via* demethylating H3K27me2, and KDM7A inhibitor TC-E 5002 can significantly reduce cell viability in cisplatin-resistant bladder cancer cell lines.^336^ PHF8, also named KDM7B, can enhance the expression of FOXA2, which is essential for the development of neuroendocrine prostate cancer (NEPC) through demethylation modification (Table 3).^337^ Besides, PHF8 and HER2 can interact with each other in breast cancer, thus promoting the resistance to trastuzumab and other anti-HER2 drugs by regulating the expression of IL6 (Fig. 11).^338^

*Other jumonji containing proteins and drug resistance*: In addition to the above six families, some other jumonji containing proteins, particularly JARID2, play an important role on drug resistance and tumor recurrence. Although JARID2 does not have demethylase activity, it can affect the cell stemness by increasing the expression of Notch1 and promoting resistance of non-small lung cell to cisplatin (Table 3).^339^ Interestingly, JARID2 was downregulated in glioblastoma, and JARID2 overexpression could reduce CCND1 expression and promote apoptosis after treated with temozolomide.^340^

##### Histone arginine demethylases and drug resistance

**Fig. 12:**
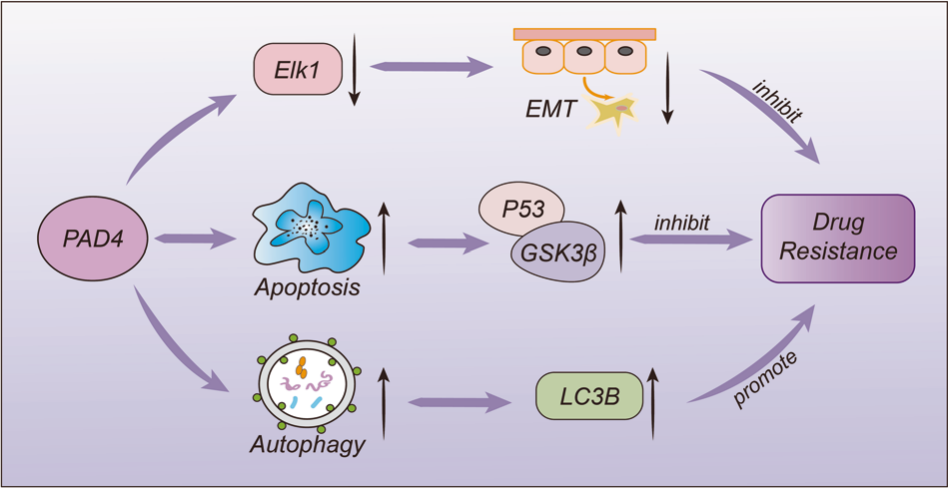
The opposite roles of histone arginine demethylase PAD4 in drug resistance. PAD4 can not only inhibit the production of drug resistance through regulating EMT and apoptosis, but also promote autophagy and enhance drug resistance

PAD4, which can transform arginine into citrulline, is also closely related to drug resistance. In gefitinib-resistant NSCLC, the expression of PAD4 is significantly downregulated. On the contrary, PAD4 overexpression can inhibit EMT by decreasing Elk1 expression, thereby inhibiting drug resistance.^341^ Zhou et al. demonstrated that PAD4 was decreased in adriamycin-resistant breast cancer. Overexpression of PAD4 promoted apoptosis and increased the expression of p53 and GSK3β so as to increase the sensitivity of cells to doxorubicin (Fig. 12).^342^ Interestingly, PAD4 can also induce autophagy and increase LC3B expression in hepatocellular carcinoma, thus promoting cell chemoresistance (Fig. 12).^343^ Although PAD4 has different influence on drug resistance in different cancers, this evidence suggest that targeting PAD4 might be a feasible strategy to decrease drug resistance.

#### Targeting histone demethylases for overcoming drug resistance

Studies have shown that drug resistance is an important cause of poor prognosis and relapse in cancer patients, and the relationship between histone demethylation and cancer resistance has attracted extensive attention.^344^ Evidences have suggested that histone demethylase inhibitors or other molecules targeting histone demethylation can effectively control drug resistance. In this section, we will discuss the effects of histone demethylase inhibitors and small interfering RNA on drug resistance.

##### Targeting LSD family for overcoming drug resistance

LSD1 is closely related to poor prognosis and can promote drug resistance in many cancer cells. Many studies have shown that LSD1 inhibitors can increase the sensitivity of cells and reverse drug resistance.^241,345–347^ Arborinine, a natural product isolated from *G. parva*, is a reversible LSD1 inhibitor, which can inhibit the proliferation of adriamycin-resistant gastric cancer cells by increasing H3K4me1 and H3K9me1/2 levels and inhibiting EMT (Table 4).^345^ The LSD1 inhibitor HCI-2509 significantly inhibited colony formation, decreased the expression of C-MYC protein, and blocked the cell cycle to inhibit cell proliferation of PC3 and DU145 cells and docetaxel-resistant prostate cancers (Table 4).^346^ Additionally, GSK2879552 and pargyline can decrease the number of Lgr5^+^ CSCs significantly to enhance the sensitivity of drug-resistant cells to sorafenib.^267^ GSK2879552 can significantly reduce the level of LINC01134 in HepG2 and MHCC97H cells and increase drug sensitivity (Table 4).^271^ Similarly, Etani et al. first reported that NCL1, a highly selective LSD1 inhibitor, can inhibit cell proliferation and autophagy in a dose-dependent manner both in vivo and in vitro (Table 4).^347^ More interestingly, targeting the PELP1-KDM1 axis with NCL1 can inhibit the transcription activation of LSD1 mediated ERα target genes and reverse drug resistance to tamoxifen or letrozole.^348^ Furthermore, 2-PCPA^89^ and SP2059^100^ can reverse the drug resistance of breast cancer and NSCLC. To conclude, targeting LSD1 or in combination with LSD1 inhibitors is an effective method to reverse drug resistance. In contrast, the development of LSD2 inhibitors and the capability of reversing drug resistance are poorly studied.

**Table 4:** Histone demethylase inhibitors and siRNA for overcoming drug resistance

Regulatory factor	Enzyme	Cancer type	Mechanism	Reference
Arborinine	LSD1	Gastric cancer	Inhibit the expression of LSD1 and EMT	^345^
HCI-2509	LSD1	Prostate cancer	Inhibit colony-forming ability and reduce the expression of C-MYC to inhibit proliferation	^346^
GSK2879552	LSD1	Hepatoma	Reduce the level of LINC01134	^271^
NCL1	LSD1	Breast cancer	Inhibit LSD1 and cell proliferation and autophagy	^347,348^
NSC636819	KDM4A	Lung, breast, and prostate cancer	Increase the expression of DR5 and TRAIL and activate exogenous apoptosis	^349^
ML324	KDM4B	Prostate cancer	Inhibit the demethylation activity of KDM4B	^298^
JIB-04	KDM4A	leukemia	Increase H3K36me3 level and restore the sensitivity to cytosine arabinoside	^350^
ZINC33576	KDM5A	Breast Cancer	Inhibit the demethylation activity of KDM5A to H3K4 and block the cell in G1 phase	^352^
PBIT	KDM5B	Non-small cell carcinoma	Inhibit lung cancer stem cells like phenotype to regulate cell sensitivity	^308^
CPI-455	KDM5A	APL	Promote cell differentiation induced by all-trans retinoic acid	^358^
GSK-J4	KDM6A; KDM6B	Colorectal cancer	Inhibit the expression of Notch2 and increase the sensitivity to oxaliplatin	^329^
	KDM2B	Hepatoma	Reduce the self-renewal ability of stem cells	^282^
Circ0006168	KDM3C	ESCC	Positively regulate the expression of KDM3C	^290^

##### Targeting the JmjC domain-containing proteins for overcoming drug resistance

Increasing evidences have demonstrated that regulating the expression of KDM family can affect the emergence of cell drug resistance, some small-molecule inhibitors and small interfering RNAs (siRNA) capable of overcoming drug resistance have been reported. NSC636819, a KDM4A/B inhibitor, can increase the expression of DR5 and TRAIL and activate exogenous apoptosis by TRAIL-DR5, leading to tumor growth inhibition in vivo and in vitro.^349^ The KDM4B inhibitor ML324 inhibited the growth of enzalutamide-resistant tumors (Table 4).^298^ Mar et al. showed that the KDM4A inhibitor JIB-04 can increase H3K36me3 levels and restore the sensitivity of leukemia cells to cytosine arabinoside (Fig. 9).^350^ Besides, JIB-04 can inhibit KDM6B and play a synergistic role with GSK-J4 in inhibiting the proliferation of TMZ-resistant cells (Table 4).^351^ Recently, Yang et al. reported that the KDM5A inhibitor ZINC33576 can effectively inhibit the demethylation activity of KDM5A, block the cell in G1 phase, and induce cell senescence, thereby playing an anti-proliferative effect (Table 4).^352^ The KDM5A inhibitor YUKA1 significantly inhibited the proliferation of gefitinib-resistant EGFR-mutant lung cancer cells.^353^ In NSCLC, PBIT, an inhibitor of KDM5B, can reduce KDM5B expression and inhibit lung cancer stem cells (LSCS) like phenotype to increase cell sensitivity to cisplatin and doxorubicin.^308^ When PBIT was used, the cell survival rate of cisplatin-resistant canine melanoma cell line decreased, indicating that inhibiting KDM5B can reduce the resistance of cells to cisplatin (Fig. 10).^313^ Interestingly, CPI-455 was identified as a specific inhibitor of the KDM5 family.^354,355^ CPI-455 can decrease the survival of erlotinib-tolerant cells after lethal drug exposures in multiple cell culture models and significantly inhibited the proliferation of temozolomide-resistant glioblastoma cells.^356,357^ In addition, CPI-455 can inhibit KDM5A to sensitize NB4 cells to all-trans retinoic acid which induced cell differentiation in acute promyelocytic leukemia (APL).^358^

The KDM6 inhibitor GSK-J4 is effective in reversing resistance of various cancers, such as colorectal cancer, chondrosarcoma, and lymphoma (Table 4). GSK-J4 can inhibit the expression of Notch2, thereby increasing the sensitivity of colorectal cancer to oxaliplatin.^329^ Significantly, GSK-J4 can cooperate with 5-FU to inhibit the proliferation of colorectal cancer and reduce cell stemness, thus increasing sensitivity.^359^ In addition, Lhuissier et al. found that chondrosarcoma is resistant to both radiotherapy and chemotherapy. And the combination of GSK-J4 and cisplatin can significantly inhibit the proliferation of chondrosarcoma cells.^360^ Moreover, when GSK-J4 is combined with vincristine and doxorubicin, it can significantly promote the apoptosis of DLBCL.^332^ Interestingly, in addition to KDM6A and KDM6B, GSK-J4 also inhibited KDM2B, reduced the expression of KDM2B, and increased the H3K36 methylation levels, thereby reducing the self-renewal ability of stem cells and increased the sensitivity of cells to drugs.^282^

Besides, siRNA can also regulate the expression of KDM family proteins. Circ0006168, a cyclic RNA having a complementary miR-194-5p binding sequence with KDM3C, can positively regulate the expression of KDM3C, and its downregulation can inhibit the growth of tumors and enhance the sensitivity of cells to paclitaxel by decreasing the expression of KDM3C (Table 4).^290^ In conclusion, the KDM family is an important regulator of tumor resistance. Except for the inhibitory role of KDM5C in cancer resistance, the other KDM family members often promote tumor resistance. Therefore, targeting the KDM family proteins could be an effective strategy to overcome drug resistance. Of note, although there exist many PAD4 inhibitors, their association with drug resistance has not been described.

## Conclusions and perspectives

Malignant tumors seriously threaten human health, and the inevitable emergence of drug resistance in tumors has posed continuous challenges to chemotherapeutics. The mechanisms for tumor resistance are complex, and epigenetic regulation could mediate cancer resistance. In this review, we mainly discuss the association of DNA methylation, non-coding RNAs, histone acetylation, and histone methylation with tumor resistance. Evidence has suggested that epigenetic regulators may act as promoters or inhibitors in different tumors. Many epigenetic regulators are overexpressed in tumors and contribute to drug resistance mainly by inhibiting apoptosis, inducing autophagy and EMT, enhancing stemness, and activating multiple signaling pathways including the PI3K/Akt, Notchand NF-κB pathways, etc. In addition, p53 repression is also an important mechanism for epigenetic regulators to promote tumor resistance. As a tumor suppressor, p53 expression can be suppressed by lncRNA Miat, KAT2A, LSD2, KDM3A, and KDM5C, which in turn promote drug resistance. For these regulators that promote tumor progression and drug resistance, targeted inhibition can effectively increase tumor sensitivity to chemotherapy. Interestingly, there are also some regulators that can act as a double-edged sword, such as DNA methyltransferase DNMT1, DNA demethylases TET1 and TET2, HAT p300, and histone demethylases KDM5C. Therefore, further elucidation of their specific roles in different cancers is urgently required to achieve the goal of targeting epigenetics to overcome drug resistance precisely.

Clearly, histone demethylases can regulate tumorigenesis in a manner dependent or independent of their demethylase activity. Currently, more than 20 histone demethylases have been discovered, and these demethylases are involved in the development of drug resistance through multiple mechanisms. As mentioned above, KDM5C showed the opposite effects in different cancers. For example, in colon cancer and clear cell renal cell carcinoma, overexpression of KDM5C can inhibit drug resistance, while in other tumors, such as gastric cancer and CRPC, KDM5C promotes tumor growth and drug resistance. However, other demethylases, which are always highly expressed in various cancers, often promote the development of drug resistance. Until now, many histone demethylase inhibitors have been developed, and several have already entered clinical trials, providing a direction for the development of targeted inhibitors. However, in addition to LSD1 inhibitors, almost all other demethylase inhibitors are in preclinical studies. Unfortunately, although many histone demethylase inhibitors and siRNA have been reported to be able to overcome drug resistance, these inhibitors mentioned above have not entered the clinical stage. Additionally, the issue of how to improve the selectivity and specificity of epigenetic inhibitors to avoid disturbing the overall epigenetic status should also be considered.

Notably, when used alone or in combination with other drugs, these inhibitors can often increase the efficacy of chemotherapeutics. Considering the interaction between some epigenetic regulators to co-regulate tumor development, the combination of two or more inhibitors with different targets may exert better effects to reverse drug resistance. Therefore, the development of multiple target inhibitors, proteolysis targeting chimeric (PROTAC) molecules, allosteric inhibitors, and those inhibitors regulating non-enzymatic functions may also provide new strategies for reversing tumor resistance. Overall, the regulatory mechanisms of epigenetics in cancer resistance provide a molecular basis for personalized cancer therapy, and targeting epigenetics holds great promise in overcoming tumor resistance.
